# Optimizing exercise prescriptions for improving neuropsychiatric symptoms in older adults with MCI or dementia: a dose-response meta-analysis

**DOI:** 10.3389/fnagi.2026.1978798

**Published:** 2026-10-28

**Authors:** Xiang Yu, Xinyu Chao, Canbo Wang, Yuanfeng Liu

**Affiliations:** 1 Party Committee Office & Principal Office, Chengdu University of Technology, Chengdu, Sichuan, China; 2 Postdoctoral Research Station for Management Science and Engineering, Xi’an University of Technology, Xi’an, Shaanxi, China; 3 Sports Institute, Chengdu University of Technology, Chengdu, Sichuan, China

**Keywords:** cognitive impairment, dementia, exercise, neuropsychiatric symptoms, older adults

## Abstract

**Background:**

Mild cognitive impairment (MCI) and dementia are common among older adults and are often accompanied by neuropsychiatric symptoms, which negatively affect quality of life and functional independence. Although exercise is a promising non-pharmacological intervention, the effective dose ranges for different exercise modalities in improving neuropsychiatric symptoms remain unclear. This study aimed to examine the dose-response relationships between different exercise modalities and neuropsychiatric symptoms among older adults with MCI or dementia.

**Methods:**

Exercise dose was calculated as the product of duration, frequency, and intensity, expressed as metabolic equivalent of task minutes per week (MET-min/week). Bayesian network and dose-response meta-analyses were conducted to compare the effects of different exercise modalities and characterize their dose–response relationships.

**Results:**

Nineteen studies involving 1587 older adults with MCI or dementia were included. Overall exercise showed a U-shaped dose-response association with improvements in neuropsychiatric symptoms, with the greatest effect observed at approximately 490 MET-min/week (SMD = −0.51, 95% CrI: −0.91, −0.08). When stratified by exercise modality, aerobic exercise (8 arms), resistance training (4 arms), and mind-body exercise (7 arms) were significantly associated with improvements in neuropsychiatric symptoms, although their effective and optimal doses varied across modalities. The doses associated with the model-identified greatest effects were 460 MET-min/week for aerobic exercise (SMD = −0.53, 95% CrI: −0.76, −0.24; very low certainty evidence), 470 MET-min/week for resistance training (SMD = −0.49, 95% CrI: −0.76, −0.17; very low certainty evidence), and 490 MET-min/week for mind–body exercise (SMD = −0.63, 95% CrI: −0.94, −0.28; low certainty evidence). No significant moderating effects were identified in network meta-regression analyses.

**Conclusion:**

Aerobic exercise, resistance training, and mind–body exercise showed distinct dose–response relationships for improving neuropsychiatric symptoms in older adults with MCI or dementia. However, given the very low to low certainty of evidence, these findings should be interpreted cautiously. Further high-quality randomized controlled trials are warranted to confirm these findings.

**Systematic review registration:**

https://www.crd.york.ac.uk/PROSPERO/view/CRD420261486763, identifier CRD420261486763.

## 1 Introduction

Mild cognitive impairment and dementia represent different stages along the continuum of cognitive disorders in older adults. Mild cognitive impairment is characterized by measurable cognitive decline without substantial impairment in daily functioning, whereas dementia involves more severe cognitive deterioration accompanied by functional limitations (Gates et al., 2019; Petersen et al., 2014). Although mild cognitive impairment does not always progress to dementia, affected individuals have a substantially increased risk of conversion, highlighting this stage as an important window for early intervention (Langa and Levine, 2014; Petersen, 2004). Dementia, as the advanced stage of cognitive decline, has become a major global health challenge, with approximately 50 million individuals affected worldwide and the number projected to exceed 130 million by 2050 (Prince et al., 2013; World Health Organization, 2021). This growing burden of cognitive disorders poses substantial social, economic, and healthcare challenges for individuals, families, and societies. Therefore, early and effective interventions targeting both mild cognitive impairment and dementia are essential to delay cognitive decline and improve health outcomes among older adults.

Neuropsychiatric symptoms, also referred to as behavioral and psychological symptoms of dementia, are common non-cognitive manifestations of cognitive disorders, including apathy, depression, anxiety, psychosis, agitation, aggression, and sleep disturbances (Kales et al., 2015). These symptoms frequently occur in individuals with mild cognitive impairment and dementia, with approximately 80%–90% of people with dementia experiencing neuropsychiatric symptoms during disease progression (Aalten et al., 2005; Cerejeira et al., 2012). Neuropsychiatric symptoms are associated with accelerated cognitive decline, impaired daily functioning, poorer quality of life, and increased risks of severe dementia and mortality (Karttunen et al., 2011; Lyketsos et al., 1997), highlighting the need for effective interventions. Pharmacological treatments, including antidepressants, anticonvulsants, antipsychotics, and mood stabilizers, are commonly used but may be limited by adverse effects and associated medical risks (Cerejeira et al., 2012). Consequently, increasing attention has been directed toward non-pharmacological interventions as safer and more sustainable approaches. Exercise, defined as “planned, structured, and repetitive physical activity aimed at improving or maintaining physical fitness” (Chodzko-Zajko et al., 2009), has been recognized as a promising intervention in dementia care (Livingston et al., 2020).

Recent evidence suggests that exercise-based interventions, particularly when combined with usual care, may effectively alleviate neuropsychiatric symptoms compared with other non-pharmacological strategies (Yin et al., 2024). Furthermore, several network meta-analyses have compared the effects of different exercise modalities on neuropsychiatric symptoms among individuals with dementia or mild cognitive impairment, consistently supporting the beneficial effects of exercise, although the magnitude of benefits may vary across exercise types (Hu et al., 2025; Kong et al., 2025). However, an important evidence gap remains: existing studies have not yet established specific recommendations regarding the effective dose ranges of different exercise modalities or provided sufficient quantitative evidence to guide the optimization of exercise prescriptions. This limitation restricts the translation of current evidence into practical, dose-specific exercise strategies. Exercise dose, including intensity, duration, and frequency, represents a fundamental component of exercise prescription. Therefore, identifying effective dose ranges for different exercise modalities is important for informing the optimization of individualized exercise strategies and improving neuropsychiatric symptoms in older adults with mild cognitive impairment or dementia.

To fill this evidence gap, a Bayesian dose-response network meta-analysis provides a suitable analytical framework, as it enables the simultaneous evaluation of multiple exercise modalities and doses while accounting for between-study heterogeneity (Mawdsley et al., 2016). This approach offers two important advantages: (1) it allows the identification of effective and optimal doses for individual exercise modalities; and (2) beyond conventional network meta-analysis, which primarily focuses on comparing intervention effects, it enables the integrated assessment and ranking of different exercise modalities across dose levels, thereby providing more clinically informative evidence for exercise prescription. Therefore, this study aimed to characterize the dose–response relationships between different exercise modalities and neuropsychiatric symptoms among older adults with mild cognitive impairment or dementia based on evidence from randomized controlled trials (RCTs). The findings may provide quantitative evidence to inform the development of individualized, dose-specific exercise strategies for improving neuropsychiatric symptoms in this population.

## 2 Methods

### 2.1 Protocol and registration

This systematic review and network meta-analysis was prospectively registered in PROSPERO (registration number: CRD420261486763) and performed following the PRISMA-NMA reporting guidelines (Hutton et al., 2015).

### 2.2 Search strategy

Multiple electronic databases, including PubMed, Web of Science, Embase, and the Cochrane Central Register of Controlled Trials, were systematically searched from inception to July 15, 2026, without language limitations. The search strategy combined free-text keywords and MeSH terms related to the population (cognitive impairment or dementia), intervention (exercise), outcome (neuropsychiatric symptoms), and study design (randomized controlled trials), using the Boolean operator “AND.” In addition, the reference lists of pertinent publications were screened manually to identify potentially eligible studies that may not have been retrieved through database searches. The complete search strategies for each database are presented in Supplementary Content 1.

### 2.3 Eligibility criteria

Eligibility criteria were established using the PICOS format (Population, Intervention, Comparison, Outcomes, Study Design) to minimize clinical bias in study selection (Tacconelli, 2010). Table 1 summarizes the eligibility criteria for included studies based on the PICOS framework.

**TABLE 1:** Eligibility criteria formulated based on the PICOS framework.

PICOS	Inclusion criteria	Exclusion criteria
Population	Studies involving older adults aged ≥ 60 years with mild cognitive impairment or dementia diagnosed according to established and validated diagnostic criteria (e.g., NIA-AA criteria, DSM-5 criteria, or other recognized clinical guidelines)	Studies including participants with major organ diseases (e.g., cardiovascular disease, cancer, or severe neurological disorders) were excluded to improve population homogeneity
Intervention	Structured exercise interventions (e.g., aerobic exercise, resistance exercise, mind–body exercise, or combined aerobic and resistance training) with clearly reported intervention characteristics, including exercise type, frequency, duration, and session length, were eligible for inclusion to enable evaluation of exercise dose–response relationships	Studies in which exercise was combined with additional co-interventions (e.g., nutritional supplementation or dietary modification) were excluded unless these co-interventions were equally implemented in both the intervention and control groups. This approach was adopted to minimize potential confounding effects and ensure that the observed outcomes were attributable primarily to exercise
Comparison	A control group (e.g., usual care) was required. For direct comparison studies, the control group could include any form of exercise intervention, including different intensities within the same exercise modality	Other interventions beyond exercise or usual care
Outcomes	Outcomes assessing neuropsychiatric symptoms measured using validated instruments, such as the Neuropsychiatric Inventory (NPI) and other established neuropsychiatric symptom scales	Studies that did not assess neuropsychiatric symptoms, lacked pre- and post-intervention outcome data, or did not provide sufficient statistical information for effect size calculation were excluded
Study design	Only randomized controlled trials were included; for studies reporting the same trial, only the one with the largest sample size was included	Non- randomized controlled trials, such as quasi-experimental and observational studies

### 2.4 Outcomes

Neuropsychiatric symptoms were assessed using validated instruments, including the Neuropsychiatric Inventory (NPI), Neuropsychiatric Inventory-Clinician Rating Scale (NPI-C), Neuropsychiatric Inventory-Nursing Home version (NPI-NH), and Neuropsychiatric Inventory Questionnaire (NPI-Q). Lower scores on these scales indicate fewer or less severe neuropsychiatric symptoms, reflecting better neuropsychiatric status (Cummings et al., 1994).

### 2.5 Data extraction and coding

Two reviewers (YX and CXY) independently performed data extraction using a standardized Excel template. Extracted information included study characteristics (e.g., author, publication year, and geographical location), participant characteristics (e.g., age and sample size), intervention characteristics (e.g., exercise modality, frequency, duration, and intervention length), comparator details (e.g., control type and sample size), and outcome information (e.g., reported findings). Mean values and standard deviations (SDs) of neuropsychiatric symptom scores were extracted at baseline and post-intervention for both exercise and control groups. Any disagreements during the extraction process were resolved through discussion between the reviewers and, when necessary, adjudicated by a third reviewer (WCB). Mean change scores were calculated for both intervention and control groups as the difference between pre- and post-intervention values to quantify changes in neuropsychiatric symptoms. When the standard deviations (SDs) of change scores were unavailable, they were estimated from baseline and post-intervention SDs using a correlation coefficient of 0.5, representing a pragmatic intermediate assumption in the absence of reported pre-post correlations (Higgins et al., 2019). When SDs were not directly reported, they were derived from available statistical information, including confidence intervals, *p*-values, or *t*-values. If sufficient information was unavailable, the study authors were contacted for clarification or data provision. As dose-response analyses require SEs as the measure of uncertainty, SDs were converted to SEs using the following formula:


$$S\,E=\frac{S\,D}{\sqrt{N}}$$

where *N* represents the sample size.

The data were cross-checked for accuracy by the reviewers, and a third author (WCB) verified the final dataset.

To perform the dose-response network meta-analysis, interventions were classified according to a three-level framework. At the first level, all interventions were broadly categorized into two groups: ‘Exercise’ and ‘Control.’ At the second level, exercise interventions were classified according to the exercise modality reported in the original studies, including ‘Aerobic Exercise’ (e.g., walking, running, cycling; AE), ‘Resistance Training’ (e.g., resistance bands and weight training; RT), ‘Mind–Body Exercise’ (e.g., dancing, yoga and tai chi; MBE), and ‘Combined Aerobic and Resistance Training’ (AE-RT). At the third level, exercise doses were quantified based on weekly exercise volume, calculated as the product of exercise duration, frequency, and intensity, and expressed as metabolic equivalent of task (MET)-minutes per week. Warm-up and cool-down periods were excluded when calculating total exercise duration if their durations were explicitly reported in the primary studies. When these periods were not separately reported, the reported exercise duration was used as provided without additional assumptions or adjustments. For interventions involving progressive increases in exercise duration throughout the intervention period, the average exercise duration per session was calculated across the intervention period and used for dose estimation. For example, when session duration increased from 40 min during the first 4 weeks to 50 min during the subsequent 4 weeks of an 8-week intervention, a duration of 45 min per session was used for dose estimation. Exercise intensity was coded according to the 2024 Adult Physical Activity Guidelines, considering the exercise modality and intensity indicators reported in the original studies (e.g., percentage of maximal heart rate and rating of perceived exertion) (Herrmann et al., 2024). This guideline provides MET values covering a wide range of exercise types, which were sufficient for allocating intensity in our analysis.

Based on the estimated MET-min/week, each intervention was assigned to the nearest predefined dose category. The predefined categories ranged from 0 (usual care) to 1100 MET-min/week and included 0, 200, 250, 300, 350, 400, 450, 550, 600, 750, 900, and 1100 MET-min/week. For each intervention, the absolute difference between its estimated dose and each predefined category was calculated, and the intervention was assigned to the category with the smallest difference. Thus, the maximum allowable distance was half the interval between adjacent categories. This approach was used to maintain adequate connectivity within the evidence network while minimizing discrepancies between the estimated and categorized doses.

When exercise attendance was reported, the estimated dose was adjusted according to the attendance rate to reflect the actual exercise exposure. For example, if an intervention involved moderate-intensity cycling at 7 METs for 40 min per session, three times per week, the estimated dose was 840 MET-min/week (7 × 40 × 3). If the average attendance rate was 90%, the adjusted dose was 756 MET-min/week (840 × 0.90), which was assigned to the nearest predefined category of 750 MET-min/week. Detailed procedures and examples for calculating exercise dose are provided in Supplementary Content 2.

### 2.6 Statistical analysis

#### 2.6.1 Dose-response network meta-analysis

As the included studies used different outcome measurement instruments, standardized mean differences (SMDs) were calculated based on changes from baseline to post-intervention assessments in both the exercise and control groups. Negative SMD values indicated greater reductions in neuropsychiatric symptom scores in the exercise group compared with the control group, reflecting beneficial effects of exercise. The dose-response relationships between exercise dose and different exercise modalities were examined using the ‘MBNMAdose’ package in R (version 4.4.3; www.r-project.org). Following the default specifications of the ‘MBNMAdose’ package, vague (non-informative) prior distributions were assigned to minimize the influence of prior assumptions and allow parameter estimates to be primarily informed by the observed data.

Before conducting the dose-response analysis, we evaluated the fundamental assumptions of network meta-analysis, including network connectivity, consistency of evidence, and incoherence across comparisons (Dias et al., 2013; Donegan et al., 2013; Higgins et al., 2012). Global consistency was assessed by comparing the consistency model with the unrelated mean effects (UME) model using the deviance information criterion (DIC), residual deviance, and between-study standard deviation. Local inconsistency between direct and indirect evidence was evaluated using node-splitting analyses. A *p*-value > 0.05 was considered to indicate no statistically significant evidence of inconsistency. Second, multiple dose-response functions were evaluated to characterize the relationship between exercise dose and treatment effects, including Emax, restricted cubic spline, log-linear, and quadratic models. Model fit was compared using several goodness-of-fit indicators, including the deviance information criterion (DIC), residual standard deviation, model complexity, and residual deviance. Following previous dose–response network meta-analysis studies, the model with the lowest DIC value was considered to provide the best fit and was selected for subsequent estimation of dose–response relationships (Cheng et al., 2026; Zhang et al., 2025, 2026a,b). Considering the anticipated between-study heterogeneity, a random-effects model was applied to account for variability across studies. The estimated dose–response parameters were used to identify exercise doses associated with significant improvements in neuropsychiatric symptoms and to estimate the relative ranking of different exercise modalities and dose levels based on their probability of achieving beneficial effects (Pedder et al., 2019). In the Bayesian dose-response analyses, statistical significance was determined when the 95% credible interval (CrI) of the estimated effect did not include zero. Finally, based on the resulting effective dose ranges, we formulated exercise prescriptions with reference to standardized adult physical activity intensity classification tables (Herrmann et al., 2024).

#### 2.6.2 Additional analyses

Network meta-regression analyses were conducted to explore whether study-level characteristics influenced the estimated intervention effects. Potential effect modifiers included mean age (years), intervention length (weeks), and proportion of female participants (%). Regression coefficients with 95% credible intervals (CrIs) were estimated to assess the associations between these covariates and neuropsychiatric symptom outcomes; a CrI including zero was interpreted as indicating insufficient evidence of an association. All network meta-regression models were implemented using the R package ‘MBNMAdose’.

Sensitivity analyses were performed by excluding studies judged to have a high risk of bias to examine the robustness of the primary findings. In addition, studies involving participants with mild cognitive impairment were excluded to evaluate whether the inclusion of populations with less severe cognitive impairment influenced the estimated effects. Potential publication bias and small-study effects were assessed through visual inspection of funnel plots and Begg’s and Egger’s tests (Begg and Mazumdar, 1994; Lin et al., 2018). A visually symmetrical funnel plot and a *p*-value > 0.05 were considered indicative of no substantial evidence of publication bias.

### 2.7 Risk of bias and certainty of evidence

Risk of bias was evaluated using the Cochrane Risk of Bias 2 (RoB 2) tool, which assesses five domains: the randomization process, deviations from intended interventions, missing outcome data, measurement of the outcome, and selection of the reported result. Each study was categorized as having a low risk of bias, some concerns, or a high risk of bias (Sterne et al., 2019). Two authors (YX, CXY) independently performed the assessment, with disagreements resolved by a third author (WCB).

The certainty of evidence for the network estimates was assessed using the confidence in network meta-analysis (CINeMA) web application, which evaluates six domains: within-study bias, reporting bias, indirectness, imprecision, heterogeneity, and incoherence (Nikolakopoulou et al., 2020). Evidence confidence was graded as high, moderate, low, or very low.

## 3 Results

### 3.1 Study selection

The database search identified 9719 records from four electronic databases. After removing 1832 duplicates, 7887 records underwent title and abstract screening, resulting in the exclusion of 7798 irrelevant records. Subsequently, 89 full-text articles were assessed for eligibility, of which 73 were excluded based on the predefined inclusion and exclusion criteria. Consequently, 16 studies met the eligibility criteria and were included in the systematic review. An additional three studies were identified through reference screening of relevant reviews, resulting in a final inclusion of 19 studies for the quantitative analysis. The study selection process is illustrated in Figure 1.

**FIGURE 1:**
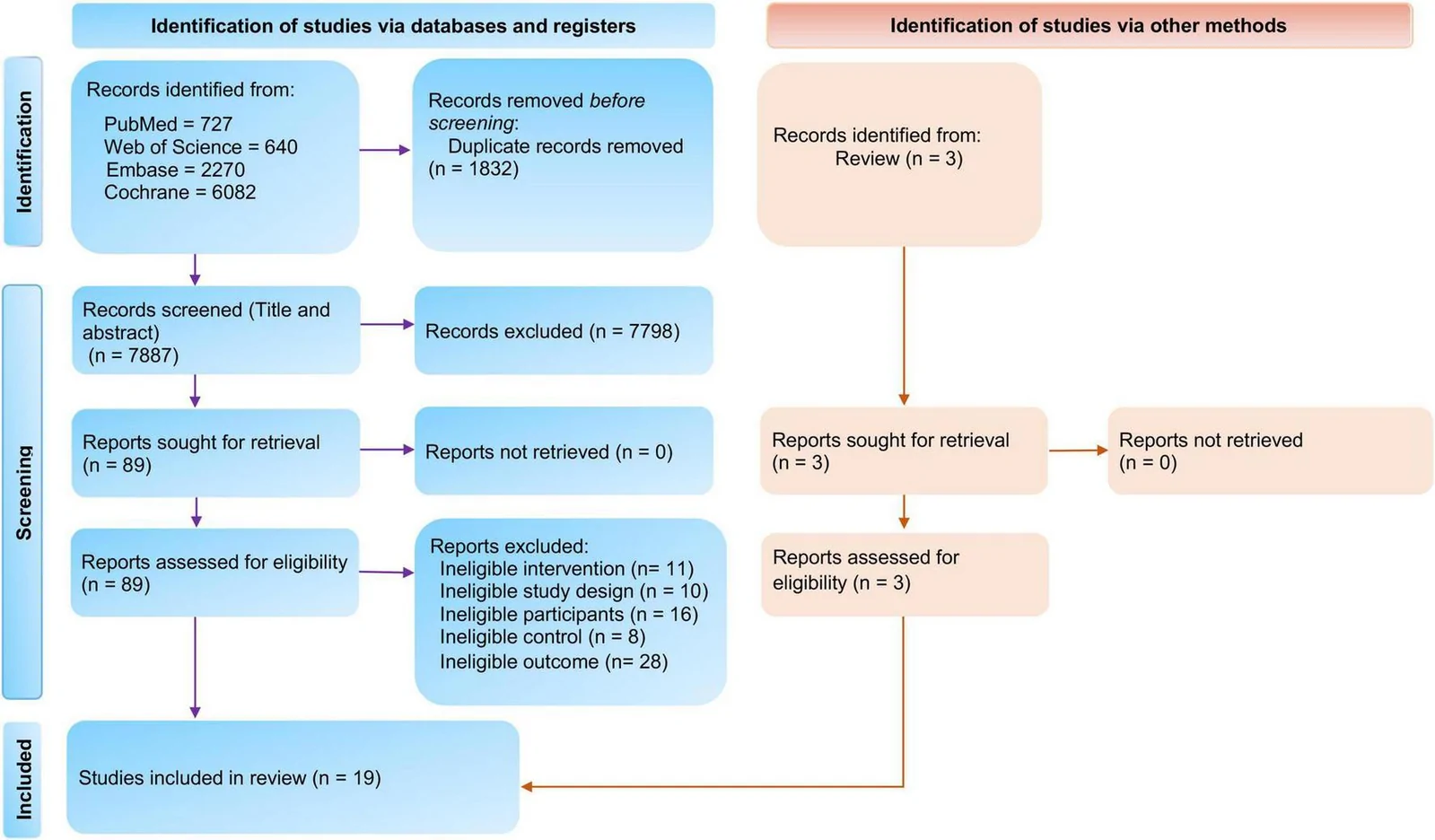
Screening and selection process of studies for the systematic review.

### 3.2 Study characteristics

A total of 1587 older adults with mild cognitive impairment or dementia participated in the included studies, with a mean age of 78.4 years. Females accounted for the majority of participants, representing 63.83% of the total sample. Regarding cognitive status, Ayari et al. (2023) included participants with both dementia and mild cognitive impairment, two studies focused on cognitive decline or mild cognitive impairment, and the remaining studies involved participants with dementia, predominantly Alzheimer’s disease. Regarding comparisons between intervention and control groups (CG), 7 studies compared.

AE with CG, 4 compared RT with CG, 7 compared MBE with CG, and 3 compared AE-RT with CG. Additionally, 1 study directly compared MBE with AE. All non-exercise control groups received usual care without structured exercise interventions. The prescribed exercise doses ranged from 200 to 1100 MET-min/week, with intervention length ranging from 2 to 65 weeks. Reported adherence rates varied from 53.8% to 100%. All included studies assessed neuropsychiatric symptoms using instruments from the Neuropsychiatric Inventory family, including the Neuropsychiatric Inventory (NPI), Neuropsychiatric Inventory-Clinician Rating Scale (NPI-C), Neuropsychiatric Inventory-Nursing Home version (NPI-NH), and Neuropsychiatric Inventory Questionnaire (NPI-Q). The included studies were conducted across multiple geographical regions, including Asia, Europe, and the Americas. No adverse events with a clear causal relationship to the exercise interventions were reported in the included studies. Detailed characteristics of the included studies are provided in Supplementary Content 3.

### 3.3 Risk of bias

Overall, one study was rated as having a high risk of bias, primarily due to a high proportion of missing outcome data. Seventeen studies were judged as having some concerns regarding risk of bias, primarily due to insufficient reporting of the randomization process, including limited details on sequence generation and allocation concealment; lack of blinding of participants and personnel, which may have introduced deviations from the intended interventions; inadequate information on the blinding of outcome assessors and the methods used to measure outcomes; and the absence of trial registration or prespecified analysis protocols, making it difficult to determine whether the reported results were selected from multiple outcome measurements or analyses. One study was classified as having a low risk of bias across all domains. Detailed risk-of-bias assessment results are provided in Supplementary Content 4.

### 3.4 Dose-response network meta-analysis

#### 3.4.1 Hypothesis testing and model selection

No evidence was found to suggest that the dose-response network meta-analysis violated the assumptions of connectivity (Figure 2 and Supplementary Content 5.1), consistency, or inconsistency (Supplementary Content 5.2). Ultimately, the quadratic function, which had the lowest DIC (178.2), was selected as the best-fitting approach among the four candidate models: Emax (DIC = 178.6), restricted cubic spline (DIC = 182.9), log-linear (DIC = 179.8), and quadratic (DIC = 178.2) (Supplementary Content 5.3).

**FIGURE 2:**
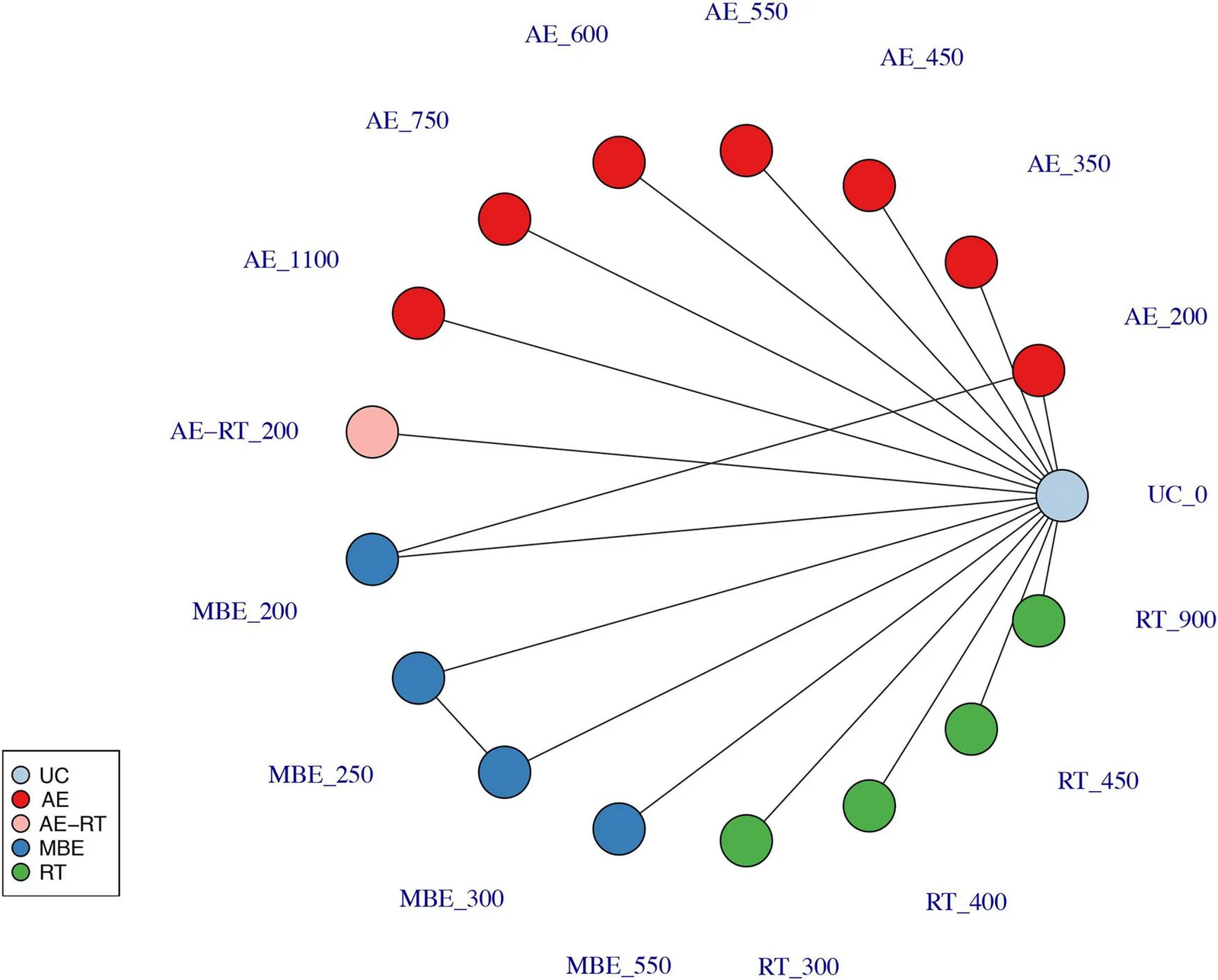
Dose-level network. Line thickness represents the number of studies available for comparison. The first value represents the specific intervention, and the second value corresponds to the dose (MET-min/week). AE: Aerobic Exercise; MBE: Mind-Body Exercise; RT: Resistance Training; AE-RT: Aerobic and Resistance Training; UC: Usual Care.

#### 3.4.2 Dose-response relationship between exercise and neuropsychiatric symptoms

Table 2 summarizes the results of the dose-response network meta-analysis. Our analysis identified a U-shaped dose–response relationship between total exercise dose and improvements in neuropsychiatric symptoms among older adults with mild cognitive impairment or dementia. The effective dose range was 300–640 MET-min/week, with the greatest improvement observed at an optimal dose of 490 MET-min/week (SMD = −0.51, 95% CrI: −0.91, −0.08; Figure 3 and Supplementary Content 5.4). Sensitivity analysis excluding studies judged to have a high risk of bias showed a slight change in the effective dose range (270–640 MET-min/week), while the optimal dose remained unchanged at 490 MET-min/week (Supplementary Content 5.5). When studies involving participants with mild cognitive impairment were excluded, neither the effective dose range nor the optimal dose changed substantially; however, the estimated beneficial effect of exercise was slightly increased (SMD = −0.53, 95% CrI: −0.95, −0.07).

**TABLE 2:** Summary of dose-response network meta-analysis.

Category	Group	Arms^a^	Effective dose (MET-min/week)^b^	Optimal dose (MET-min/week)^c^	Standard Mean difference and 95% CrI (optimal dose)
Overall results	All exercise modalities	22	300–640	490	−0.51 (−0.91, −0.08)
Stratified by exercise modality	AE-RT	3	NA	NA	NA
	AE	8	190–720	460	−0.53 (−0.76, −0.24)
	RT	4	220–650	470	−0.49 (−0.76, −0.17)
	MBE	7	170–550	490	−0.63 (−0.94, −0.28)
Sensitivity analyses	Excluding high risk bias studies	21	270–640	490	−0.53 (−0.92, −0.11)
Sensitivity analyses	Excluding cognitive impairment studies	19	300–490	490	−0.53 (−0.95, −0.07)

Effective dose and the dose associated with the optimal effect were derived from the dose–response model

*^a^*The number of exercise arms included in each analysis.

*^b^*Model-identified effective dose (MET-min/week), calculated as exercise intensity × duration × frequency.

*^c^*Model-identified optimal dose (MET-min/week), calculated as exercise intensity × duration × frequency. NA: Not Applicable (no statistically significant effect observed). AE: aerobic exercise; AE-RT: aerobic and resistance training; MBE: Mind-Body Exercise; RT: resistance training; MET-min/week: metabolic equivalent of task minutes per week.

**FIGURE 3:**
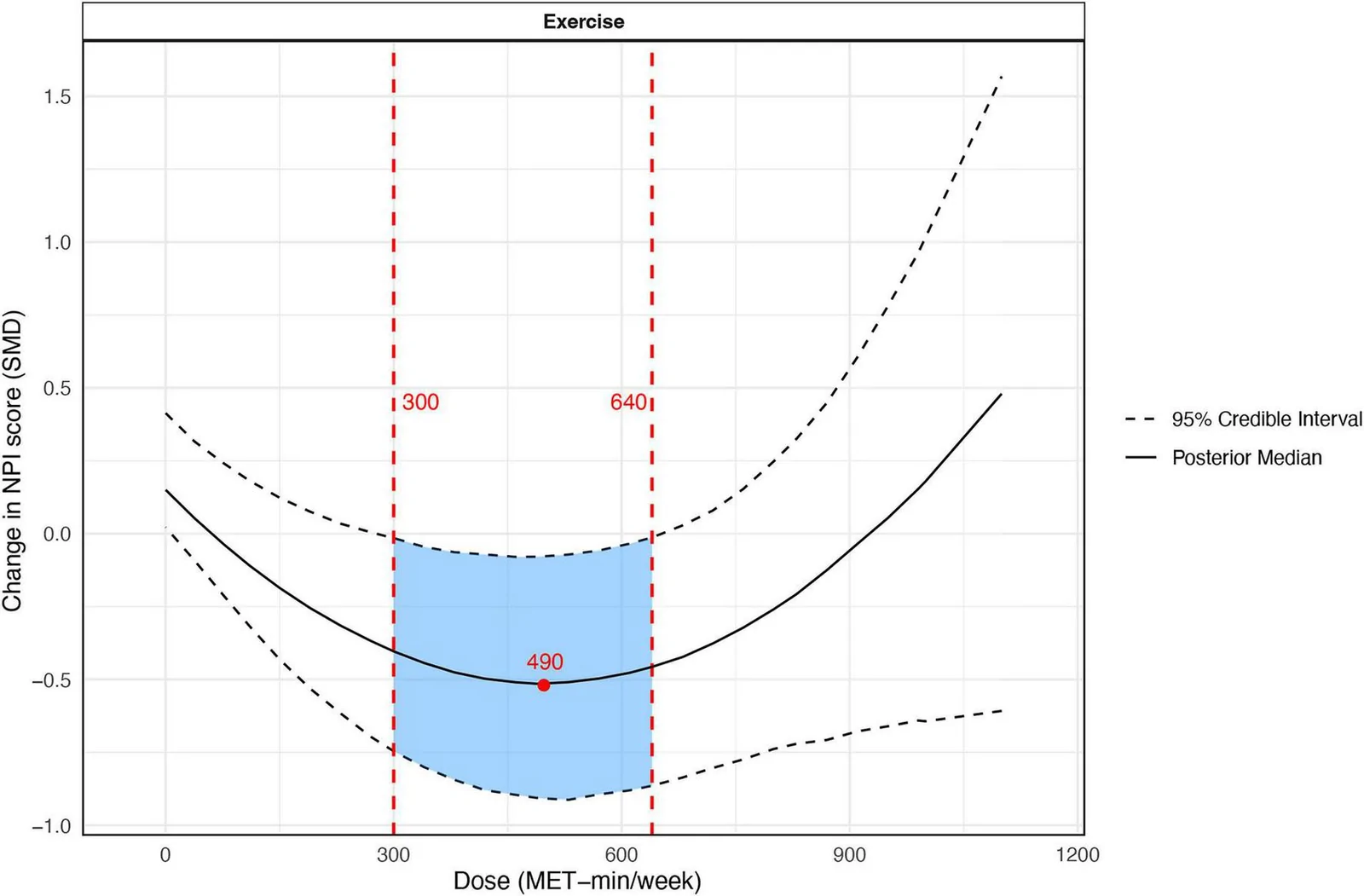
Dose–response association between total exercise dose (MET-min/week) and improvements in neuropsychiatric symptoms among older adults with mild cognitive impairment or dementia. Negative standardized mean differences (SMD) indicate improvements in neuropsychiatric symptoms. The red dashed lines represent the minimum and maximum effective doses (300–640 MET-min/week), the blue shaded area indicates the significant region, and the red dots represent the optimal dose points (490 MET-min/week). MET-min/week: Metabolic Equivalent of Task minutes per week; SMD: Standardized Mean Difference; NPI: Neuropsychiatric Inventory.

Figure 4 illustrates the dose-response associations between different exercise modalities and neuropsychiatric symptoms. Significant dose-response relationships were identified for AE, RT, and MBE, whereas no significant association was observed for AE-RT. For AE, the effective dose range was 190–720 MET-min/week, with the greatest model-estimated effect at 460 MET-min/week (SMD = −0.53, 95% CrI: −0.76, −0.24). For RT, the effective dose range was 220–650 MET-min/week, with the greatest model-estimated effect at 470 MET-min/week (SMD = −0.49, 95% CrI: −0.76, −0.17). For MBE, the effective dose range was 170–550 MET-min/week, with the greatest model-estimated effect at 490 MET-min/week (SMD = −0.63, 95% CrI: −0.94, −0.28).

**FIGURE 4:**
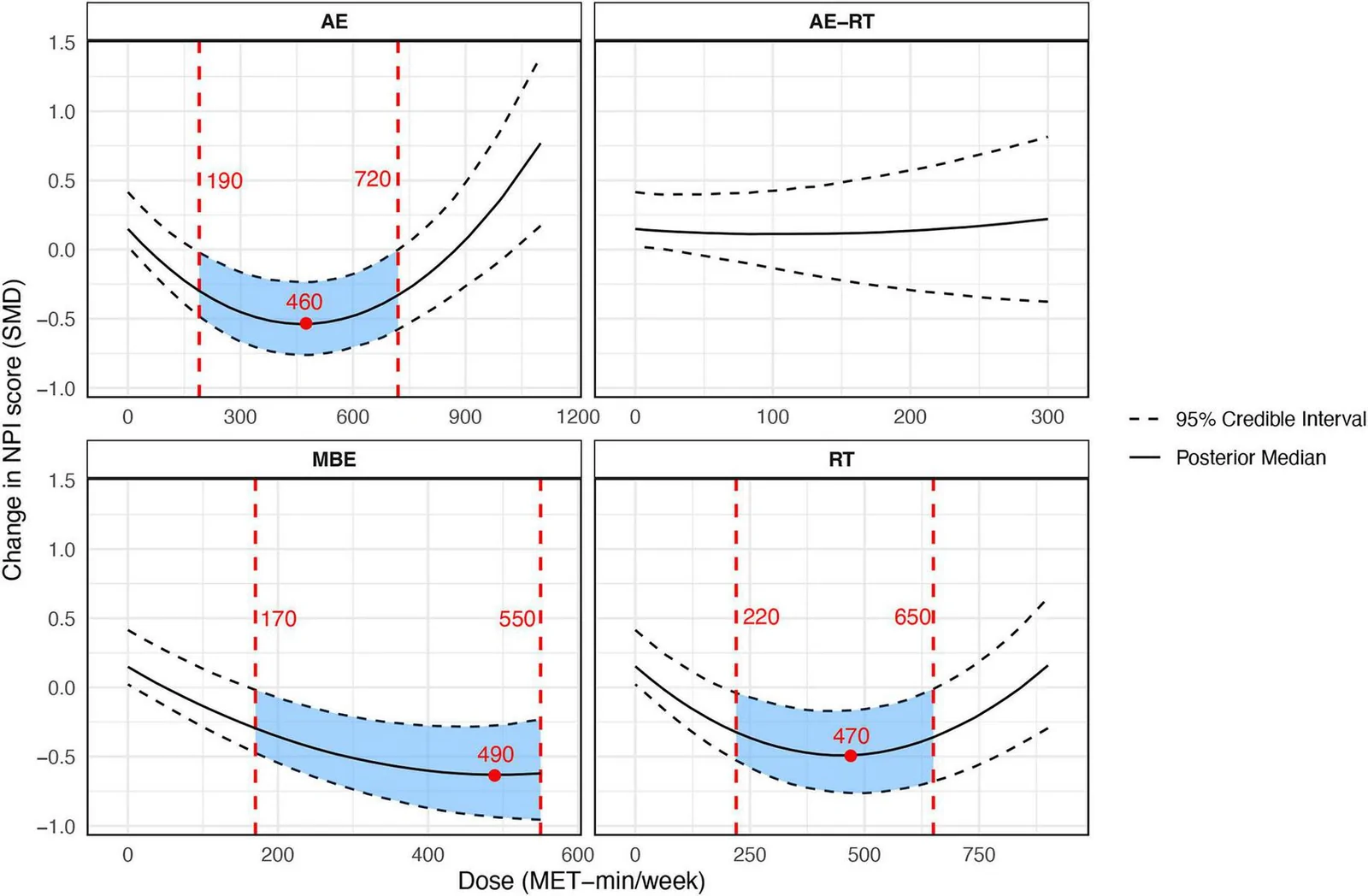
Dose–response association between different exercise dose (MET-min/week) and improvements in neuropsychiatric symptoms among older adults with mild cognitive impairment or dementia. The red dashed lines represent the minimum and maximum effective doses, the blue shaded area indicates the significant region, and the red dots represent the optimal dose points. AE-RT did not show a statistically significant effect across the evaluated dose range. AE: Aerobic Exercise; MBE: Mind-Body Exercise; RT: Resistance Training; AE-RT: Aerobic and Resistance Training; MET-min/week: Metabolic Equivalent of Task minutes per week; SMD: Standardized Mean Difference; NPI: Neuropsychiatric Inventory.

The overall predicted ranking of exercise modalities and doses indicated that MBE at 490 MET-min/week was the most effective intervention-dose combination (Supplementary Content 5.6). Network meta-regression analyses showed that none of the evaluated covariates, including age, sex, and intervention length, significantly modified the effects of exercise interventions on neuropsychiatric symptoms (Supplementary Content 6).

### 3.5 Translation of exercise doses

Table 3 presents the approximate translation of the model-derived effective exercise doses into weekly exercise durations across the seven exercise types for improving neuropsychiatric symptoms in older adults with mild cognitive impairment or dementia. Overall, approximately 25–205 min of exercise per week, depending on the exercise type, corresponded to the effective dose ranges identified in the present analysis.

**TABLE 3:** Translation of model-derived exercise doses.

Outcome	Exercise modality	Energy expenditure (MET)	Recommended accumulation (min/week)	Certainty of evidence (grade)
Neuropsychiatric symptoms (NPI-family instruments)	Walking^a^	3.5 (code: 17160)	55–205	Very low
	Cycling^b^	7.0 (code: 01016)	25–105	Very low
	Jogging/running^c^	4.8 (code: 12025)	40–150	Very low
	Resistance training^d^	3.5 (code: 02054)	60–185	Very low
	Yoga	2.3 (code: 02175)	75–240	Low
	Tai chi / qigong	3.3 (code: 15670)	50–165	Low
	Dance^e^	4.8 (code: 02005)	35–115	Low

Only exercise categories with a statistically supported effective in Table 2 are included. Walking, cycling, jogging/running, yoga, tai chi/qigong, and dance were classified based on the original exercise modalities reported in the included studies. Weekly exercise accumulation was calculated by dividing the prescribed MET-min/week by the estimated energy expenditure of each exercise modality and rounding to the nearest whole minute. Activity-specific MET values and codes were obtained from the 2024 Adult Compendium of Physical Activities.

*^a^*Walking for pleasure.

*^b^*Cycling, self-selected moderate pace.

*^c^*Jogging, in place.

*^d^*Resistance (weight) training, multiple exercises, 8–15 repetitions at varied resistance.

*^e^*The dance value uses representative low-impact aerobic dance. 3–6 MET represents moderate-intensity activity, and >6 MET represents high-intensity activity, serving as a reference for absolute exercise intensity. Exercise prescription recommendations are based on the currently available evidence and should be interpreted as exploratory and hypothesis-generating; they may be refined as additional evidence emerges. Certainty ratings were taken from the current study’s CINeMA assessment and are category-level ratings. Values were rounded to the nearest minute; CINeMA: confidence in network meta-analysis; NPI: neuropsychiatric inventory.

### 3.6 Publication bias and certainty of evidence

Detailed assessments of publication bias are presented in Supplementary Content 7. Visual inspection of the funnel plot showed no apparent asymmetry, and neither Egger’s nor Begg’s tests indicated significant small-study effects (*p* > 0.05), suggesting that the results were unlikely to be substantially influenced by publication bias.

The CINeMA assessment indicated that the certainty of evidence across the four exercise interventions ranged from very low to low. The main factors contributing to downgrading the certainty of evidence were within-study bias, heterogeneity, imprecision, and incoherence in the network estimates. Notably, all four exercise interventions were downgraded for within-study bias (Supplementary Content 8).

## 4 Discussion

### 4.1 Principal findings and implications

This study synthesized the comparative effects of different exercise modalities and doses on neuropsychiatric symptoms among older adults with mild cognitive impairment or dementia. We identified a U-shaped dose–response relationship between overall exercise dose and improvements in neuropsychiatric symptoms, with the greatest benefit observed at approximately 490 MET-min/week. When examined by exercise modality, AE, MBE, and RT were all associated with significant symptom improvements, although the certainty of evidence was rated as very low to low. MBE demonstrated the largest improvement at approximately 490 MET-min/week, while AE and RT achieved their maximal effects at 460 and 470 MET-min/week, respectively. These findings indicate that the dose required to optimize neuropsychiatric benefits may vary across exercise modalities, emphasizing the need to consider both exercise characteristics and dose when developing individualized exercise prescriptions for older adults with cognitive impairment. Network meta-regression analyses found no significant associations between study-level characteristics and treatment effects, suggesting that the observed benefits were not substantially influenced by potential effect modifiers. Furthermore, sensitivity analyses yielded consistent results, supporting the stability of the primary findings.

These findings have important clinical and research implications. First, they may assist healthcare professionals in developing more individualized exercise strategies to improve neuropsychiatric symptoms in older adults with mild cognitive impairment or dementia. Second, the modality-specific and dose-specific estimates identified in this study may provide quantitative evidence to inform the design of future intervention trials aimed at further evaluating and validating the observed dose–response relationships and strengthening the evidence base for exercise prescription in this population.

### 4.2 Interpretation and comparison with existing literature

A U-shaped dose–response pattern was observed, although this finding should be interpreted cautiously given the relatively sparse data at higher doses. This pattern suggests that higher exercise doses were not necessarily associated with greater benefits and is broadly consistent with a previous cross-sectional study that examined the dose–response association between exercise and cognitive function in older adults (Wang et al., 2024). Gallardo-Gómez et al. (2022) also used a similar Bayesian model-based dose–response approach and identified 724 MET-min/week as the dose associated with the minimal clinically important difference in cognitive function among older adults. In our study, the lower effective dose identified may partly reflect differences in the outcome measures and study populations. First, owing to the heterogeneity of the neuropsychiatric symptom scales used across the included studies, we did not prespecify a minimal clinically important difference, and our effective dose estimates were therefore based on the modeled statistical effects rather than a predefined clinical threshold. Second, our study specifically focused on older adults with mild cognitive impairment or dementia, who may have greater functional limitations and a higher symptom burden than the general older population, potentially resulting in greater responsiveness to exercise. In our study, insufficient exercise doses (e.g., <300 MET-min/week) may not provide an adequate stimulus to induce meaningful improvements, whereas excessive exercise exposure may reduce adherence, or limit recovery, potentially attenuating the beneficial effects of exercise (Symons et al., 2023). This pattern may reflect the complex dose-response characteristics of exercise. However, the mechanisms underlying this U-shaped association remain unclear and warrant further investigation. Additionally, the limited number of studies and sparse data points at higher exercise doses may have contributed to the observed non-linear relationship. The beneficial effects of exercise on neuropsychiatric symptoms may involve multiple neurobiological mechanisms. Exercise enhances cerebral blood flow and supports brain function by improving oxygen and glucose delivery. It also regulates neurotransmitter systems, including serotonin and dopamine pathways, which play important roles in mood and behavioral regulation (Chapman et al., 2013; Knöchel et al., 2012). Furthermore, exercise promotes the release of neurotrophic factors, such as brain-derived neurotrophic factor, insulin-like growth factor-1, and vascular endothelial growth factor, which support neuroplasticity and synaptic function (Coelho et al., 2013). These adaptations may contribute to improvements in neuropsychiatric symptoms among older adults with cognitive impairment.

When stratified by exercise modality, MBE showed the greatest improvement in neuropsychiatric symptoms, which differs from the findings of Kong et al. (2025), who reported resistance training as the most effective modality. This discrepancy may be partly explained by differences in study inclusion criteria, as their analysis incorporated quasi-randomized controlled trials and Chinese-language studies, potentially resulting in variations in the characteristics of the included evidence. Notably, the differences in effects among the three exercise modalities were relatively small when each was evaluated at its optimal dose. Therefore, the relative ranking of exercise modalities should be interpreted cautiously, as the preferred modality may change with the accumulation of additional high-quality evidence. The potential benefits of MBE for neuropsychiatric symptoms may be related to its combined effects on physical, psychological, and neurophysiological pathways. MBE incorporates controlled movements, breathing regulation, and attentional focus, which may enhance autonomic regulation, reduce stress responses, and improve emotional regulation (Streeter et al., 2010). In addition, these practices may influence neurotransmitter activity and neuroplasticity-related pathways, thereby contributing to improvements in anxiety, depressive symptoms, agitation, and other neuropsychiatric manifestations (Tang et al., 2009).

The beneficial effects of RT on neuropsychiatric symptoms may be partly explained by its influence on neurotrophic pathways. A recent systematic review showed that resistance training may induce greater increases in circulating brain-derived neurotrophic factor (BDNF) levels compared with aerobic exercise, interval training, and combined training (Zhou et al., 2022). BDNF, through binding with tropomyosin receptor kinase B (TrkB), plays an important role in neuronal survival, synaptic plasticity, and neurocognitive resilience, which may contribute to the regulation of neuropsychiatric symptoms in individuals with cognitive impairment. In contrast, AE may improve neuropsychiatric symptoms through complementary mechanisms, including enhanced cerebral blood flow, improved vascular function, and reduced neuroinflammation and oxidative stress (Knaepen et al., 2010). These adaptations may support brain health and emotional regulation, thereby alleviating symptoms such as anxiety, depressive symptoms, and behavioral disturbances. Finally, although AE–RT did not show a significant effect on neuropsychiatric symptoms, this finding should be interpreted cautiously due to the limited number of available studies. Given the reported benefits of combined aerobic and resistance training on cognitive function and other health outcomes in older adults, further high-quality RCTs are needed to clarify its effects on neuropsychiatric symptoms (Timmons et al., 2018; Zhang et al., 2024).

### 4.3 Clinical recommendations

The World Health Organization currently recommends 600–1200 MET-min/week of physical activity for healthy adults (Bull et al., 2020). However, for older adults with cognitive impairment, higher exercise doses may be more challenging to implement and may increase the burden associated with exercise participation. Our findings suggest that a lower overall exercise dose (300–640 MET-min/week) may be sufficient to achieve improvements in neuropsychiatric symptoms, potentially reducing the burden of exercise while maintaining beneficial effects. Importantly, the dose estimates identified in the present study are derived from dose–response modeling rather than directly demonstrated by individual RCTs. Therefore, the model-identified doses associated with the greatest estimated effects should be interpreted cautiously, as their exact locations are subject to uncertainty, particularly given the limited number and distribution of available exercise doses. Moreover, given the variation in effective dose ranges across exercise modalities, exercise strategies should not solely prioritize maximal efficacy but also consider individual preferences, feasibility, and long-term adherence. MBE may represent a practical option because of its favorable balance between effectiveness and relatively low physical demands. Regarding intervention length, as no significant moderating effect was identified for intervention length, the average length of 24 weeks observed across the included studies may represent a reasonable timeframe for achieving beneficial effects.

### 4.4 Strengths and limitations

To the best of our knowledge, this study is the first to systematically investigate the dose–response relationships between different exercise modalities and exercise doses for improving neuropsychiatric symptoms among older adults with mild cognitive impairment or dementia. A major strength of this study was the application of MET-min/week to quantify exercise dose, integrating exercise intensity, duration, and frequency into a standardized metric and enabling dose-response modeling within a unified network framework. In contrast, conventional subgroup analyses typically examine isolated components of exercise exposure, such as frequency or duration, without considering the combined contribution of different dose components. Furthermore, we performed network meta-regression analyses to explore whether study-level characteristics influenced the estimated intervention effects. By integrating direct and indirect evidence within a Bayesian framework and modeling dose-response relationships, this study extends beyond evaluating whether exercise is beneficial and provides quantitative insights into how exercise modality and dose may influence improvements in neuropsychiatric symptoms. These findings may help inform the development of more individualized exercise prescriptions and provide a quantitative reference for future clinical practice and research.

Despite these strengths, several limitations should be acknowledged. First, although MET-min/week provides a standardized approach for quantifying exercise dose by integrating intensity, duration, and frequency, this approach has inherent limitations. MET values are derived from population-based estimates of energy expenditure and may not fully reflect individual variations in age, sex, body composition, functional capacity, disease severity, or physiological responses to exercise. Furthermore, standardized MET values may not accurately capture fluctuations in exercise intensity or differences in exercise tolerance among older adults with cognitive impairment. Second, the number of eligible studies was relatively limited, and direct head-to-head comparisons among different exercise modalities were scarce, resulting in a predominantly star-shaped network with most comparisons connected through the usual-care group. Consequently, some comparative estimates relied heavily on indirect evidence and model-based estimates, which may reduce the certainty of the comparative effects. Therefore, the relative effects and rankings of different exercise modalities should be interpreted with caution. In addition, the statistical power of funnel plot-based assessments for publication bias was limited, particularly given the relatively small number of studies available for some comparisons. Excluding studies that combined exercise with other interventions may limit the generalizability of our findings, as multimodal interventions are common in clinical practice. Third, the certainty of evidence for the network comparisons was rated as very low to low. Although this does not necessarily invalidate the estimated dose-response relationships, the findings should be interpreted cautiously considering the limited evidence base, potential risk of bias, and uncertainty in the available data. In addition, clinical heterogeneity across studies, including differences in baseline physical function, cognitive status, disease severity, and intervention characteristics, may have contributed to variability in the observed effects and dose–response relationships. Therefore, the current findings should be considered exploratory and interpreted within the context of these limitations. Finally, the quadratic model used to characterize the dose–response relationships may be sensitive to the distribution of available dose data, particularly when observations are unevenly distributed across the dose range. The limited number of studies at higher dose levels may also reduce the stability and precision of the estimated dose–response curves. Therefore, these model-derived findings should be interpreted cautiously. Future well-designed RCTs with larger samples, standardized outcome assessments, and direct comparisons of different exercise doses are warranted to validate the observed dose–response relationships and refine exercise dose recommendations for managing neuropsychiatric symptoms in older adults with cognitive impairment.

## 5 Conclusion

This study systematically evaluated the comparative effects of different exercise modalities and doses on neuropsychiatric symptoms among older adults with mild cognitive impairment or dementia. We identified a U-shaped dose-response relationship between overall exercise dose and improvements in neuropsychiatric symptoms, with the model identifying approximately 490 MET-min/week as the dose associated with the greatest estimated effect. When stratified by exercise modality, AE, MBE, and RT were associated with significant improvements in neuropsychiatric symptoms, although the effective dose ranges varied across modalities.

These findings may help inform the development of individualized, dose-specific exercise strategies for improving neuropsychiatric symptoms in older adults with mild cognitive impairment or dementia. However, the identified dose estimates are derived from dose–response modeling, and the model-identified dose associated with the greatest estimated effect remains subject to uncertainty. Given the very low to low certainty of evidence, these findings should be interpreted cautiously and considered exploratory. Further high-quality RCTs are needed to validate these findings and refine exercise dose recommendations.

## Data Availability

The original contributions presented in the study are included in the article/Supplementary material, further inquiries can be directed to the corresponding author.
