# Supplementary material for: Optimizing exercise prescriptions for improving neuropsychiatric symptoms in older adults with MCI or dementia: a dose-response meta-analysis
Source: Front Aging Neurosci. 2026 Oct 28;18:1978798. doi: 10.3389/fnagi.2026.1978798 (PMC13751367; doi:10.3389/fnagi.2026.1978798)
Supplement: Supplementary file 1 [file Data_Sheet_1.pdf]

Supplementary content

1. SEARCH STRATEGY 2
1.1 PUBMED 2
1.2 WEB OF SCIENCE 3
1.3 EMBASE 3
1.4 COCHRANE 4
2. EXERCISE INTERVENTION CLASSIFICATION AND WEEKLY DOSE CODING 7
3. STUDY CHARACTERISTICS 10
4. RISK OF BIAS 15
5. DOSE-RESPONSE NETWORK META-ANALYSIS 17
5.1 NETWORK CONNECTIVITY 17
5.2 CONSISTENCY AND INCOHERENCE 17
5.3 DOSE-RESPONSE MODEL SELECTION 20
5.4 DOSE-RESPONSE RELATIONSHIPS 24
5.5 SENSITIVITY ANALYSIS 25
5.6 RELATIVE EFFECTIVENESS RANKING 27
6. META-REGRESSION ANALYSIS 30
7. REPORTING BIAS AND SMALL-STUDY EFFECTS 30
8. CONFIDENCE IN THE EVIDENCE USING CINEMA 31
8.1 WITHIN-STUDY BIAS 31
8.2 REPORTING BIAS 31
8.3 INDIRECTNESS 32
8.4 IMPRECISION 32
8.5 HETEROGENEITY 32
8.6 INCOHERENCE 33
8.7 OVERALL CINEMA JUDGMENT 33
REFERENCES 35

1. Search strategy

1.1 Pubmed

("Aged"[Mesh] OR "Aged, 80 and over"[Mesh] OR older adult*[Title/Abstract] OR older people[Title/Abstract] OR elderly[Title/Abstract] OR geriatric*[Title/Abstract]) AND ("Dementia"[Mesh] OR "Alzheimer Disease"[Mesh] OR "Mild Cognitive Impairment"[Mesh] OR "Neurocognitive Disorders"[Mesh] OR dementia[Title/Abstract] OR Alzheimer*[Title/Abstract] OR "mild cognitive impairment"[Title/Abstract] OR "major neurocognitive disorder*"[Title/Abstract] OR "neurocognitive disorder*"[Title/Abstract]) AND ("Exercise"[Mesh] OR "Exercise Therapy"[Mesh] OR "Motor Activity"[Mesh] OR "Physical Exertion"[Mesh] OR "Physical Fitness"[Mesh] OR "Resistance Training"[Mesh] OR "Aerobic Exercise"[Mesh] OR "Tai Ji"[Mesh] OR "Dancing"[Mesh] OR "Walking"[Mesh] OR "Yoga"[Mesh] OR exercis*[Title/Abstract] OR "exercise therapy"[Title/Abstract] OR "physical activity"[Title/Abstract] OR "physical exercise"[Title/Abstract] OR "aerobic exercise"[Title/Abstract] OR "aerobic training"[Title/Abstract] OR "resistance exercise"[Title/Abstract] OR "resistance training"[Title/Abstract] OR "strength training"[Title/Abstract] OR "endurance exercise"[Title/Abstract] OR walking[Title/Abstract] OR cycling[Title/Abstract] OR dance[Title/Abstract] OR dancing[Title/Abstract] OR "tai chi"[Title/Abstract] OR yoga[Title/Abstract] OR "finger exercise"[Title/Abstract] OR exergam*[Title/Abstract] OR "mind-body exercise"[Title/Abstract] OR multicomponent[Title/Abstract] OR "multi-component exercise"[Title/Abstract] OR "combined exercise"[Title/Abstract]) AND ("Neurobehavioral Manifestations"[Mesh] OR "Psychiatric Status Rating Scales"[Mesh] OR neuropsychiatric symptom*[Title/Abstract] OR neuropsychiatric manifestation*[Title/Abstract] OR "behavioral and psychological symptom*"[Title/Abstract] OR "behavioural and psychological symptom*"[Title/Abstract] OR BPSD[Title/Abstract] OR NPS[Title/Abstract] OR apathy[Title/Abstract] OR agitation[Title/Abstract] OR aggression[Title/Abstract] OR anxiety[Title/Abstract] OR depression[Title/Abstract] OR psychosis[Title/Abstract] OR delusion*[Title/Abstract] OR hallucination*[Title/Abstract] OR irritability[Title/Abstract] OR wandering[Title/Abstract] OR "neuropsychiatric inventory"[Title/Abstract] OR NPI[Title/Abstract] OR NPI-Q[Title/Abstract]

OR NPI-NH[Title/Abstract] OR NPI-C[Title/Abstract]) AND (Randomized Controlled Trial[Publication Type] OR Controlled Clinical Trial[Publication Type] OR randomized[Title/Abstract] OR randomised[Title/Abstract] OR randomly[Title/Abstract] OR RCT[Title/Abstract] OR RCTs[Title/Abstract] OR "randomized trial"[Title/Abstract] OR "randomised trial"[Title/Abstract] OR "controlled clinical trial"[Title/Abstract] OR "clinical trial"[Title/Abstract] OR "intervention trial"[Title/Abstract] OR trial[Title/Abstract] OR quasi-experiment*[Title/Abstract] OR nonrandomized[Title/Abstract] OR non-randomized[Title/Abstract]) NOT ("Animals"[Mesh] NOT "Humans"[Mesh])

1.2 Web of Science

TS=("older adult*" OR "older people" OR elderly OR geriatric*) AND TS=(dementia OR Alzheimer* OR "mild cognitive impairment" OR "major neurocognitive disorder*" OR "neurocognitive disorder*") AND TS=(exercis* OR "exercise therapy" OR "physical activity" OR "physical exercise" OR "aerobic exercise" OR "aerobic training" OR "resistance exercise" OR "resistance training" OR "strength training" OR "endurance exercise" OR walking OR cycling OR dance OR dancing OR "tai chi" OR yoga OR "finger exercise" OR exergam* OR "mind-body exercise" OR multicomponent OR "multi-component exercise" OR "combined exercise") AND TS=("neuropsychiatric symptom*" OR "neuropsychiatric manifestation*" OR "behavioral and psychological symptom*" OR "behavioural and psychological symptom*" OR BPSD OR NPS OR apathy OR agitation OR aggression OR anxiety OR depression OR psychosis OR delusion* OR hallucination* OR irritability OR wandering OR "neuropsychiatric inventory" OR NPI OR NPI-Q OR NPI-NH OR NPI-C) AND TS=(randomized OR randomised OR randomly OR RCT OR RCTs OR "randomized trial" OR "randomised trial" OR "controlled clinical trial" OR "clinical trial" OR "intervention trial" OR trial OR quasi-experiment* OR nonrandomized OR non-randomized)

1.3 Embase

('aged'/exp OR 'older adult*':ti,ab OR 'older people':ti,ab OR elderly:ti,ab OR geriatric*:ti,ab) AND ('dementia'/exp OR 'alzheimer disease'/exp OR 'mild cognitive impairment'/exp OR

'neurocognitive disorder'/exp OR dementia:ti,ab OR alzheimer*:ti,ab OR 'mild cognitive impairment':ti,ab OR 'major neurocognitive disorder*':ti,ab OR 'neurocognitive disorder*':ti,ab) AND ('exercise'/exp OR 'exercise therapy'/exp OR 'physical activity'/exp OR 'motor activity'/exp OR 'physical exertion'/exp OR 'resistance training'/exp OR 'aerobic exercise'/exp OR 'tai chi'/exp OR 'dance'/exp OR 'walking'/exp OR 'yoga'/exp OR exercis*:ti,ab OR 'exercise therapy':ti,ab OR 'physical activity':ti,ab OR 'physical exercise':ti,ab OR 'aerobic exercise':ti,ab OR 'aerobic training':ti,ab OR 'resistance exercise':ti,ab OR 'resistance training':ti,ab OR 'strength training':ti,ab OR 'endurance exercise':ti,ab OR walking:ti,ab OR cycling:ti,ab OR dance:ti,ab OR dancing:ti,ab OR 'tai chi':ti,ab OR yoga:ti,ab OR 'finger exercise':ti,ab OR exergam*:ti,ab OR 'mind-body exercise':ti,ab OR multicomponent:ti,ab OR 'multi-component exercise':ti,ab OR 'combined exercise':ti,ab) AND ('neurobehavioral manifestation'/exp OR neuropsychiatric symptom*:ti,ab OR neuropsychiatric manifestation*:ti,ab OR 'behavioral and psychological symptom*':ti,ab OR 'behavioural and psychological symptom*':ti,ab OR BPSD:ti,ab OR NPS:ti,ab OR apathy:ti,ab OR agitation:ti,ab OR aggression:ti,ab OR anxiety:ti,ab OR depression:ti,ab OR psychosis:ti,ab OR delusion*:ti,ab OR hallucination*:ti,ab OR irritability:ti,ab OR wandering:ti,ab OR 'neuropsychiatric inventory':ti,ab OR NPI:ti,ab OR NPI-Q:ti,ab OR NPI-NH:ti,ab OR NPI-C:ti,ab) AND ('randomized controlled trial'/exp OR 'controlled clinical trial'/exp OR randomized:ti,ab OR randomised:ti,ab OR randomly:ti,ab OR RCT:ti,ab OR RCTs:ti,ab OR 'randomized trial':ti,ab OR 'randomised trial':ti,ab OR 'controlled clinical trial':ti,ab OR 'clinical trial':ti,ab OR 'intervention trial':ti,ab OR trial:ti,ab OR quasi-experiment*:ti,ab OR nonrandomized:ti,ab OR non-randomized:ti,ab) AND [humans]/lim

1.4 Cochrane

#1 MeSH descriptor: [Aged] explode all trees

#2 MeSH descriptor: [Dementia] explode all trees

#3 MeSH descriptor: [Alzheimer Disease] explode all trees

#4 MeSH descriptor: [Mild Cognitive Impairment] explode all trees

#5 (older adult*):ti,ab,kw OR (older people):ti,ab,kw OR (elderly):ti,ab,kw OR (geriatric*):ti,ab,kw

#6 (dementia):ti,ab,kw OR (Alzheimer*):ti,ab,kw OR (mild cognitive impairment):ti,ab,kw OR (major neurocognitive disorder*):ti,ab,kw OR (neurocognitive disorder*):ti,ab,kw

#7 #1 OR #2 OR #3 OR #4 OR #5 OR #6

#8 MeSH descriptor: [Exercise] explode all trees

#9 MeSH descriptor: [Exercise Therapy] explode all trees

#10 MeSH descriptor: [Motor Activity] explode all trees

#11 MeSH descriptor: [Physical Fitness] explode all trees

#12 MeSH descriptor: [Resistance Training] explode all trees

#13 MeSH descriptor: [Aerobic Exercise] explode all trees

#14 MeSH descriptor: [Tai Ji] explode all trees

#15 MeSH descriptor: [Dancing] explode all trees

#16 MeSH descriptor: [Walking] explode all trees

#17 MeSH descriptor: [Yoga] explode all trees

#18 exercis*:ti,ab,kw OR (exercise therapy):ti,ab,kw OR (physical activity):ti,ab,kw OR (physical exercise):ti,ab,kw OR (aerobic exercise):ti,ab,kw OR (aerobic training):ti,ab,kw OR (resistance exercise):ti,ab,kw OR (resistance training):ti,ab,kw OR (strength training):ti,ab,kw OR (endurance exercise):ti,ab,kw OR walking:ti,ab,kw OR cycling:ti,ab,kw OR (dance OR dancing):ti,ab,kw OR (tai chi):ti,ab,kw OR yoga:ti,ab,kw OR (finger exercise):ti,ab,kw OR exergam*:ti,ab,kw OR (mind-body exercise):ti,ab,kw OR multicomponent:ti,ab,kw OR (multi-component exercise):ti,ab,kw OR (combined exercise):ti,ab,kw

#19 #8 OR #9 OR #10 OR #11 OR #12 OR #13 OR #14 OR #15 OR #16 OR #17 OR #18

#20 MeSH descriptor: [Neurobehavioral Manifestations] explode all trees

#21 MeSH descriptor: [Psychiatric Status Rating Scales] explode all trees

#22 (neuropsychiatric symptom*):ti,ab,kw OR (neuropsychiatric manifestation*):ti,ab,kw OR (behavioral and psychological symptom*):ti,ab,kw OR (behavioural and psychological symptom*):ti,ab,kw OR (BPSD OR NPS):ti,ab,kw OR (apathy OR agitation OR aggression):ti,ab,kw OR (anxiety OR depression OR psychosis):ti,ab,kw OR (delusion* OR hallucination* OR irritability OR wandering):ti,ab,kw OR (neuropsychiatric inventory):ti,ab,kw OR (NPI OR NPI-Q OR NPI-NH OR NPI-C):ti,ab,kw

#23 #20 OR #21 OR #22

#24 (randomized OR randomised OR randomly OR RCT OR RCTs OR trial OR placebo OR (controlled clinical trial) OR (randomized controlled trial) OR (randomised controlled trial) OR (clinical trial) OR (intervention trial) OR (quasi-experiment*) OR (nonrandomized) OR (non-randomized)):ti,ab,kw

#25 #7 AND #19 AND #23 AND #24

2. Exercise intervention classification and weekly dose coding

For MBE, including Dancing, Tai Chi, Qigong, and yoga, intensity was coded based on the reported exercise characteristics and corresponding MET values from the 2024 Physical Activity Compendium (e.g., tai chi 3.3 MET, yoga 2.3 MET). For AE, when intensity was reported as a percentage of maximal heart rate (%HRmax), exercise intensity was classified according to established ACSM guidelines: light intensity was defined as <64% HRmax, moderate intensity as 64–76% HRmax, vigorous intensity as 77–93% HRmax, and near-maximal to maximal intensity as ≥94% HRmax. These intensity categories were then matched with corresponding MET values from the 2024 Physical Activity Compendium. RT intensity followed ACSM guidelines based on %1 repetition maximum (1RM; light <50%, moderate 50–69%, vigorous ≥70–85%); if %1RM was unavailable, intensity was inferred from rating of perceived exertion (≈12–13 = moderate; 14–17 = vigorous) or repetition ranges (12–20, 8–12, 6–8 reps). When studies reported only qualitative intensity levels, such as “moderate,” we applied the ACSM absolute-intensity thresholds (light ≈ 2.0–2.9 MET, moderate ≈ 3.0–5.9 MET, vigorous ≥6.0 MET) and used the midpoint of each range (e.g., 4.5 MET for moderate, 8.0 MET for vigorous). For studies lacking specific intensity details, we assigned generic codes from the 2024 Physical Activity Compendium, selecting conservative estimates to minimize potential bias (e.g., general intensity for walking was 3.5 MET). For AE-RT, the total weekly exercise dose was calculated as the sum of the doses of each individual exercise component (e.g., aerobic, resistance, and balance exercises) included in the intervention program.

A complete dose-coding process for two representative studies is provided in Table S1, illustrating how exercise intensity, session duration, frequency, and attendance rate were incorporated to calculate the final exercise dose and assign it to the nearest predefined dose category.

- **Agent:** The agent level.

- **METs_min:** The energy expenditure per min in the study.
- **Min_session：** The duration of a single exercise intervention.
- **Frequency:** The number of times participants engaged in physical activity (times/week).
- **Exact_dose:** The exact estimated METs accumulated per week by participants (MET-min/week).
- **Dose:** The dose group by approximation (MET-min/week).
- **AE:** Aerobic exercise
- **MBE:** Mind-body exercise
- **CG:** Control group

Table S1. Dose categorization table

Study ID	Agent	MET_min	Min_session	Min_session (excluding warm-up)	Frequency	Prescribed dose	Adherence rate (%)	Exact_dose	Dose
Ayari et al. 2023	MBE	4.8	60	50	1	240	86.7	208	200
	AE	4.8	60	50	1	240	86.4	207	200
Bennett et al. 2024	MBE	4.5	35	35	2	315	87	274	250
	CG	0	0	0	0	0	0	0	0

3. Study characteristics

Table S2. Study characteristics of the included studies.

Study (Author, year)	Intervention (n)	Length (weeks)	Age, years mean (SD)	BMI mean (SD)	Adherence	Country	Frequency (week)	Duration (min)	Dose (MET-min/week)	Participants / Health Status	Adverse Events	Female percent (%)	Outcome scale
Ayari et al. 2023 [1]	MBE (n=11)	16	79.8(7.7)	23.9(2.1)	86.7% attendance; 11/11 completed (100%)	France	1	60	200	Sedentary older adults with MCI or mild dementia	NR	81.8	Neuropsychiatric Inventory-R (NPI-R)
	AE (n=12)	16	77.2(5.3)	26.3(3.5)	86.4% attendance; 12/12 completed (100%)	France	1	50	200	Sedentary older adults with MCI or mild dementia/probable AD	Baseline hypertension 5/12; baseline falls 9/12; causality NR	58.3	Neuropsychiatric Inventory-R (NPI-R)
Bennett et al. 2024 [2]	MBE (n=8)	12	NR	NR	Training attendance >=90 %; 8/8 completed (100%)	USA	2	35	250	Alzheimer's disease and related dementias (ADRD)	No adverse events (explicitly stated none occurred)	87	NPI-C (Neuropsychiatric Inventory-Clinician Rating Scale)
	CG (n=8)	12	NR	NR	8/8 completed (100%)	USA	NR	0	0	Alzheimer's disease and related dementias (ADRD)	No adverse events (explicitly stated none occurred)	NR	NPI-C (Neuropsychiatric Inventory-Clinician Rating Scale)
Budak et al. 2023 [3]	AE (n=9)	2	73.60(8.62)	NR	9/10 completed (90%)	Turkey	5	50	1100	Alzheimer's disease (CDR 1-2)	NR	44.4	Neuropsychiatric Inventory (NPI)
	CG (n=8)	2	74.90(8.11)	NR	NR	Turkey	NR	NR	0	Alzheimer's disease (CDR 1-2)	NR	87.5	Neuropsychiatric Inventory (NPI)
Cámara-Calmaestra et al. 2025 [4]	RT (n=30)	12	81.7(6.2)	26.0(3.4)	Training attendance >=91.7 % (33/36); 30/30 completed (100%)	Spain	3	55	900	Alzheimer's disease	NR	78	Neuropsychiatric Inventory Questionnaire (NPI-Q)
	CG (n=30)	12	82.3(7.6)	25.1(2.6)	30/30 completed (100%)	Spain	NR	NR	0	Alzheimer's disease	NR	74	Neuropsychiatric Inventory Questionnaire (NPI-Q)
Cancela et al. 2016 [5]	AE (n=51)	65	80.63(8.32)	NR	Training attendance ~88%; 51/73 completed (69.9%)	Spain	7	15	350	Institutionalized dementia	No exercise-related adverse events (explicitly stated none occurred)	43.8	Neuropsychiatric Inventory (NPI)
	CG (n=63)	65	82.90(7.42)	NR	63/116 completed (54.3%)	Spain	NR	NR	0	Institutionalized dementia	No exercise-related adverse events (explicitly stated none occurred)	81	Neuropsychiatric Inventory (NPI)

D'Amico et al. 2016 [6]	AE (n=30)	12	78.6(7.6)	NR	NR	UK	5	25	450	Community-dwelling dementia with clinically significant BPSD (ICD-10 dementia)	NR	53.3	Neuropsychiatric Inventory (NPI)
	CG (n=22)	12	78.4(9.07)	NR	NR	UK	NR	NR	0	Community-dwelling dementia with clinically significant BPSD (ICD-10 dementia)	NR	59.1	Neuropsychiatric Inventory (NPI)
Fleiner et al. 2017 [7]	RT (n=35)	2	80(7)	25.4(4.0)	Training attendance average 128/240 min/week (53.3%); 35/46 completed (76.1%)	Germany	12	20	450	Dementia (ICD-10), predominantly moderate (mean MMSE 18.3)	No serious adverse events (explicitly stated none occurred)	46	Neuropsychiatric Inventory (NPI)
	CG (n=35)	2	80(7)	25.6(3.9)	Training attendance average 105/120 min/week (87.5%); 35/39 completed (89.7%)	Germany	NR	NR	0	Dementia (ICD-10), predominantly moderate (mean MMSE 18.3)	Severe hyponatremia 1/39; cardiac decompensation 1/39; causality NR	49	Neuropsychiatric Inventory (NPI)
Hoffmann et al. 2016 [8]	AE (n=102)	16	69.8(7.4)	NR	Training attendance 84%; 102/107 completed (95.3%)	Denmark	3	60	750	Mild Alzheimer's disease (probable AD, NINDS-ADRDA criteria, MMSE >19)	35 adverse events (12 musculoskeletal problems, 10 dizziness, etc.); 1 atrial fibrillation possibly related; see original Table 3	47.7	Neuropsychiatric Inventory (NPI-12, caregiver-rated)
	CG (n=88)	16	71.3(7.3)	NR	88/93 completed (94.6%)	Denmark	NR	NR	0	Mild Alzheimer's disease (NINDS-ADRDA criteria, MMSE >19)	20 adverse events (see original Table 3)	38.7	Neuropsychiatric Inventory (NPI-12, caregiver-rated)
Holthoff et al. 2015 [9]	RT (n=15)	12	72.40(4.34)	23.43(2.75)	Training attendance >=75 % (at least 27/36 sessions); 15/15 completed (100%)	Germany	3	30	300	Alzheimer's disease (mild to moderate, NINCDS-ADRDA criteria, CDR stage 1-2, MMSE 20.6 +/- 6.5)	NR	53.33	Neuropsychiatric Inventory (NPI) total score
	CG (n=15)	12	70.67(5.41)	24.12(4.06)	15/15 completed (100%)	Germany	NR	NR	0	Alzheimer's disease (mild to moderate, NINCDS-ADRDA criteria, CDR stage 1-2, MMSE 20.6 +/- 6.5)	NR	46.67	Neuropsychiatric Inventory (NPI) total score
Huang et al. 2019 [10]	MBE (n=36)	40	81.9(6.0)	NR	36/40 completed (90%)	China	3	20	200	Mild dementia (DSM-IV, CDR <2)	Mild fatigue or limb soreness 5/36; no falls or	70	Neuropsychiatric Inventory (NPI)

											other adverse events; causality NR		
	CG (n=38)	40	81.9(6.1)	NR	38/40 completed (95%)	China	NR	NR	0	Mild dementia (DSM-IV, CDR <2)	No adverse events (explicitly stated none occurred)	65	Neuropsychiatric Inventory (NPI)
Lam et al. 2012 [11]	MBE (n=92)	52	77.2(6.3)	NR	92/171 completed (53.8%)	Hong Kong, China	3	30	300	At risk of cognitive decline (CDR 0.5 or amnestic MCI)	Death 1/171; fracture 1/171 (occurred outside exercise setting and unrelated to intervention); causality NR	73.1	Neuropsychiatric Inventory (NPI, Chinese version)
	MBE (n=169)	52	78.3(6.6)	NR	169/218 completed (77.5%)	Hong Kong, China	3	30	250	At risk of cognitive decline (CDR 0.5 or amnestic MCI)	Death 2/218; fracture 1/218 (occurred outside exercise setting and unrelated to intervention); causality NR	78.9	Neuropsychiatric Inventory (NPI, Chinese version)
Lazarou et al. 2017 [12]	MBE (n=66)	40	65.89(10.76)	NR	NR	Greece	2	60	550	Amnestic mild cognitive impairment (aMCI, Petersen criteria, GDS stage 3)	NR	80.3	Neuropsychiatric Inventory (NPI)
	CG (n=63)	40	67.92(9.47)	NR	NR	Greece	NR	NR	0	Amnestic mild cognitive impairment (aMCI, Petersen criteria, GDS stage 3)	NR	76.2	Neuropsychiatric Inventory (NPI)
Maltais et al. 2019 [13]	AE (n=44)	24	NR	NR	NR	France	2	60	550	Alzheimer's disease, vascular or mixed dementia (DSM-IV, MMSE <=20)	NR	NR	Neuropsychiatric Inventory-Nursing Home version (NPI-NH) 10-item scale
	CG (n=47)	24	NR	NR	NR	France	NR	NR	0	Alzheimer's disease, vascular or mixed dementia (DSM-IV, MMSE <=20)	NR	NR	Neuropsychiatric Inventory-Nursing Home version (NPI-NH) 10-item scale
Rolland et al. 2007 [14]	AE-RT (n=56)	52	82.8(7.8)	NR	Training attendance 33.2%; 56/67 completed (83.6%)	France	2	60	200	Alzheimer's disease (probable or possible AD, NINCDS-ADRDA criteria)	Falls 5; scalp wound 1; deaths 7; fractures 5; causality NR	71.7	Neuropsychiatric Inventory (NPI)

	CG (n=54)	52	83.1(7.0)	NR	54/67 completed (80.6%)	France	NR	NR	0	Alzheimer's disease (probable or possible AD, NINCDS-ADRDA criteria)	Total falls 136; fractures 2; deaths 8; causality NR	79.1	Neuropsychiatric Inventory (NPI)
Swinnen et al. 2021 [15]	AE (n=23)	8	84.7(5.6)	NR	Training attendance 82.9%; 23/28 completed (82.1%)	Belgium	3	15	200	Major neurocognitive disorder (MNCD, DSM-5, MMSE 18 +/- 4.4)	No study-related adverse events (explicitly stated none occurred)	78.3	Neuropsychiatric Inventory (NPI)
	CG (n=22)	8	85.3(6.5)	NR	Training attendance 73.7%; 22/27 completed (81.5%)	Belgium	3	15	0	Major neurocognitive disorder (MNCD, DSM-5, MMSE 17 +/- 4.2)	No study-related adverse events (explicitly stated none occurred)	77.3	Neuropsychiatric Inventory (NPI)
Swinnen et al. 2023 [16]	AE-RT (n=7)	12	81.9(8.2)	NR	Training attendance 61%; 7/9 completed (77.8%)	Belgium	3	30	250	Major neurocognitive disorder (MNCD, DSM-5)	Eye discomfort 1/7; knee pain 1/7; dizziness 1/7; causality NR	100	Neuropsychiatric Inventory (NPI)
	AE-RT (n=11)	12	84.2(5.9)	NR	Training attendance 63%; 11/14 completed (78.6%)	Belgium	3	30	200	Major neurocognitive disorder (MNCD, DSM-5)	Fall 1/11 (occurred while returning home after exercise; unrelated to intervention)	90.9	Neuropsychiatric Inventory (NPI)
Telenius et al. 2015 [17]	RT (n=75)	12	86.7(7.4)	NR	Training attendance 75%	Norway	2	55	400	Mild to moderate dementia (CDR 1 or 2)	No adverse events (explicitly stated none occurred)	74	Neuropsychiatric Inventory Questionnaire (NPI-Q)
	CG (n=72)	12	86.7(7.4)	NR	Training attendance 69%	Norway	2	55	0	Mild to moderate dementia (CDR 1 or 2)	No adverse events (explicitly stated none occurred)	74	Neuropsychiatric Inventory Questionnaire (NPI-Q)
Todri et al. 2020 [18]	MBE (n=45)	24	81.87(4.83)	NR	45/45 completed (100%)	Albania, Spain	3	40	300	Probable Alzheimer's disease (NINCDS-ADRDA criteria); mild to moderate	NR	62.2	Neuropsychiatric Inventory (NPI)
	CG (n=45)	24	80.47(5.59)	NR	45/45 completed (100%)	Albania, Spain	NR	NR	0	Probable Alzheimer's disease (NINCDS-ADRDA criteria); mild to moderate	NR	48.9	Neuropsychiatric Inventory (NPI)
Yang et al. 2015 [19]	AE (n=25)	12	72.00(6.69)	NR	25/25 completed (100%)	China	3	40	600	Mild Alzheimer's disease (MMSE 21.33 +/- 2.24)	NR	60	Neuropsychiatric Inventory Questionnaire (NPI-Q)

	CG (n=25)	12	71.92(7.28)	NR	25/25 completed (100%)	China	0	0	0	Mild Alzheimer's disease (MMSE 20.00 +/- 3.50)	NR	72	Neuropsychiatric Inventory Questionnaire (NPI-Q)

Note: Values are presented as mean (SD) unless otherwise specified. BMI is reported in kg/m². Frequency and duration refer to the reported intervention schedule. Dose is expressed as MET-min/week; NR indicates not reported.

Abbreviations: MCI: mild cognitive impairment; AE, aerobic exercise; AE-RT, aerobic plus resistance training; CG, control group; MBE, mind-body exercise; RT, resistance training; MET, metabolic equivalent of task; NR, not reported; NPI, Neuropsychiatric Inventory; NPI-C, Neuropsychiatric Inventory-Clinician Rating Scale; NPI-NH, Neuropsychiatric Inventory-Nursing Home version; NPI-Q, Neuropsychiatric Inventory Questionnaire;

4. Risk of bias

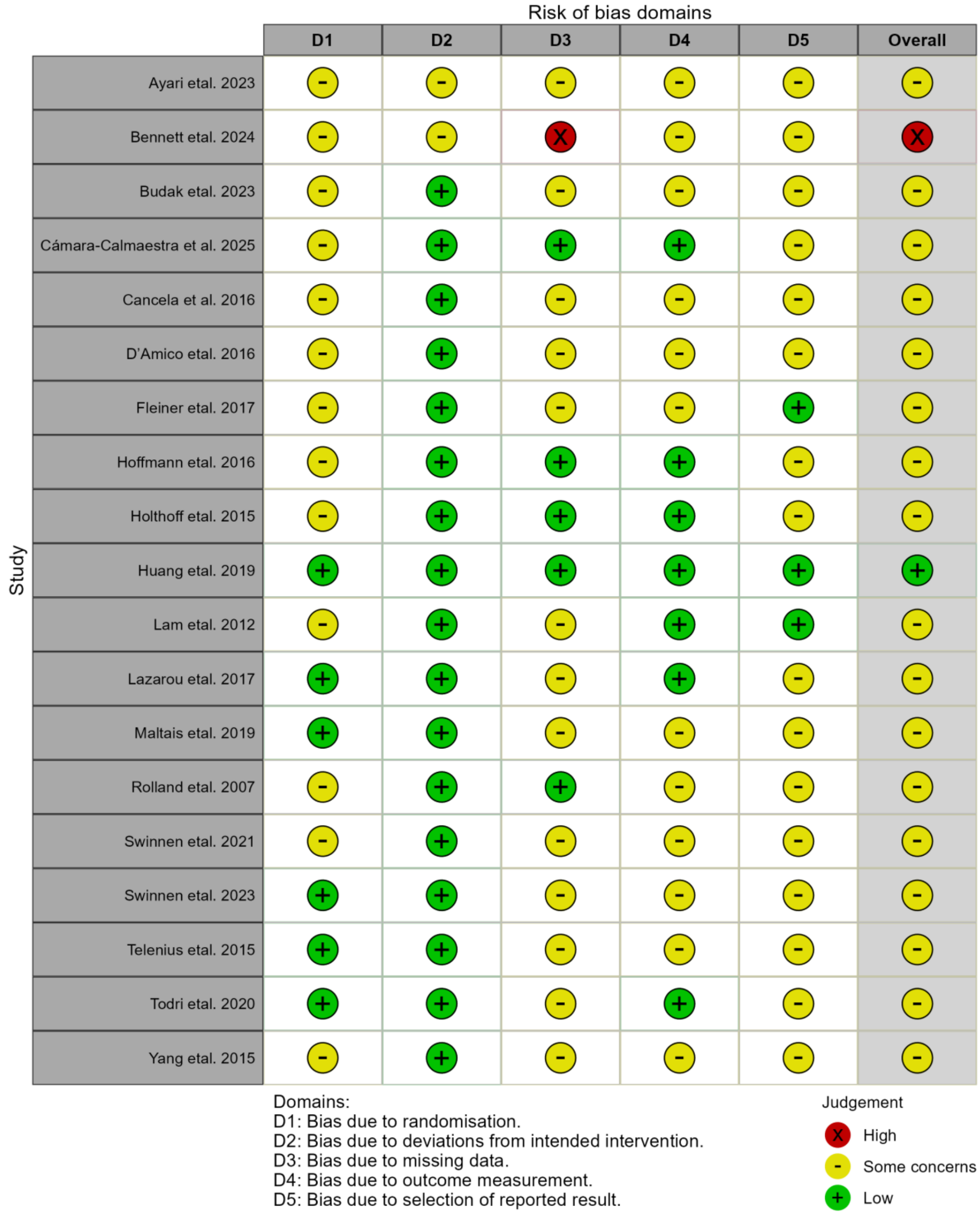

Figure S1. Risk-of-bias judgments for individual studies.

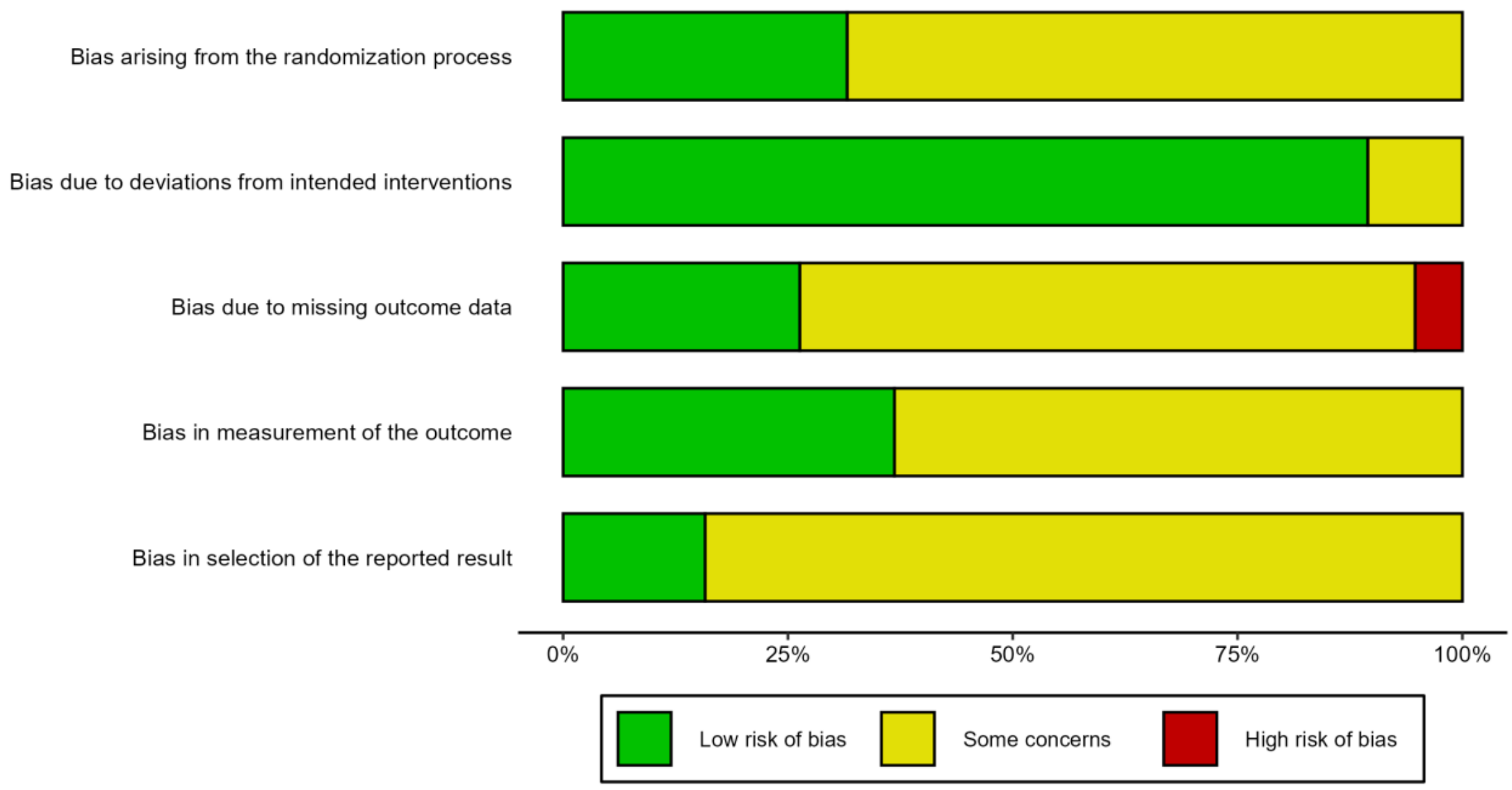

Figure S2. Summary of risk-of-bias judgments.

5. Dose-response network meta-analysis

5.1 Network connectivity

Connectivity is a fundamental assumption in network meta-analysis (NMA). In our study, the dose-based network allowed connections across both intervention modalities and dose levels, with no evidence of network disconnection observed.

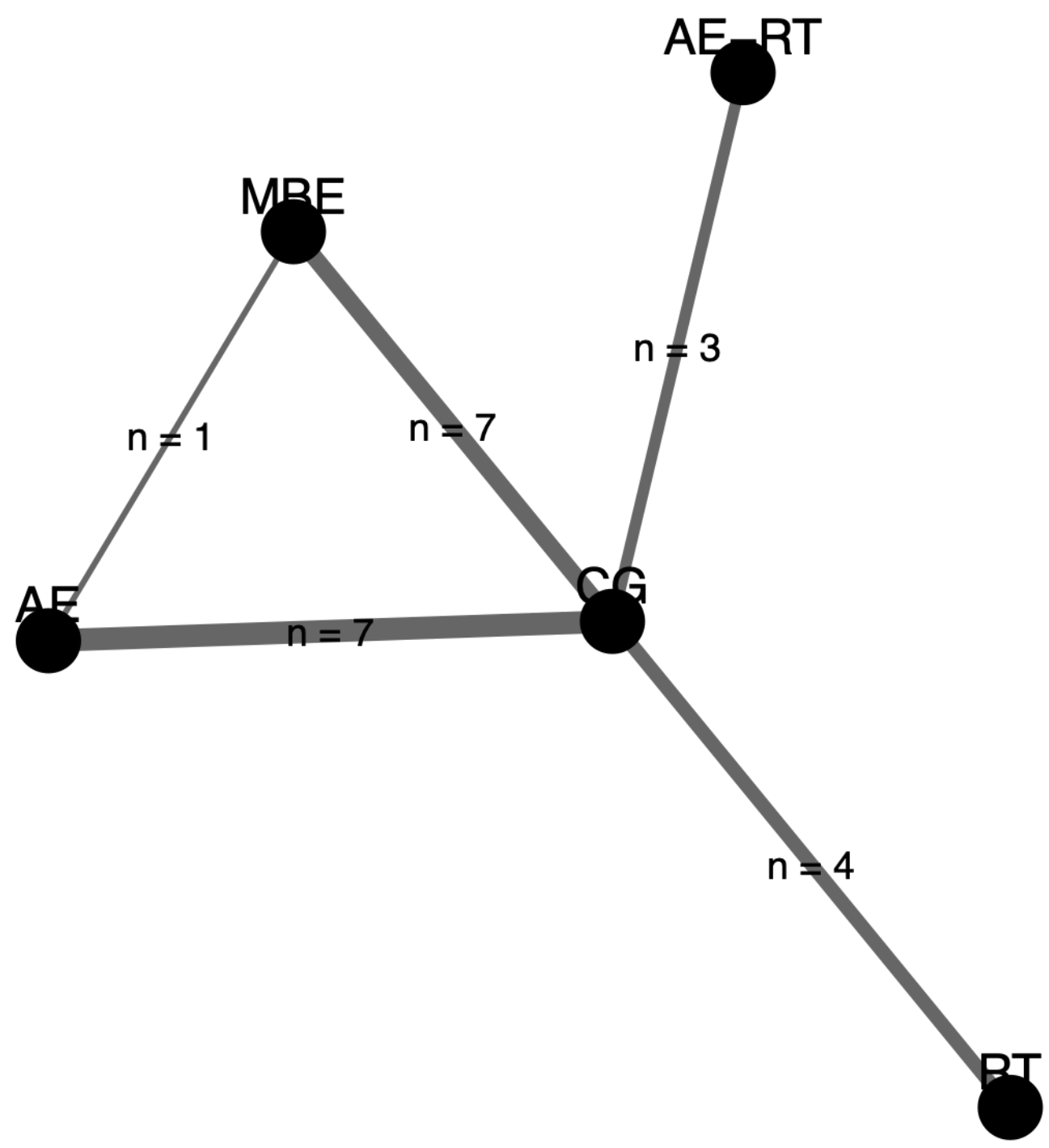

Figure S3. Exercise-level network.

Line thickness represents the number of studies available for comparison. AE: Aerobic Exercise; AE-RT:Aerobic and resistance exercise combined training; MBE: Mind-Body Exercise; RT: Resistance Training.

5.2 Consistency and inconsistency

Global consistency was assessed by comparing the fit of the consistency model with that of the unrelated mean effects (UME) model using the deviance information criterion, residual deviance, and between-study standard deviation (Table S3). Local inconsistency between direct and indirect evidence was additionally examined using node-splitting analyses (Table S4). Statistical significance was interpreted together with the magnitude and uncertainty of disagreement. A p-value >0.05 was considered to indicate no statistically significant evidence of inconsistency between direct and indirect estimates.

Table S3. Comparison of consistency and UME model fit.

Model	PD	Deviance	Residual deviance	DIC	SD
Consistent	35.7	125.207	37.975	161.3	0.649
UME	36.1	125.050	37.818	161.7	0.661

Note: UME = unrelated mean effects model; DIC = deviance information criterion; PD = effective number of parameters; SD = standard deviation

Table S4. Node-splitting analysis of inconsistency

Comparison	p-value	Median	2.5%	97.5%
MBE_300 vs MBE_250	**0.067**			
-> direct		0.217	-6.957	7.459
-> indirect		-0.071	-0.276	0.024
-> MBNMA		-0.072	-0.270	0.020
AE-RT_250 vs AE-RT_200	**0.058**			
-> direct		12.143	-2.359	27.440
-> indirect		0.020	-0.186	0.290
-> MBNMA		0.021	-0.184	0.287
MBE_200 vs AE_200	**0.447**			
-> direct		0.676	-6.306	8.146
-> indirect		-3.213	-7.720	1.259
-> MBNMA		-2.530	-6.309	0.916
RT_900 vs Placebo_0	**0.712**			
-> direct		-2.185	-9.326	5.317
-> indirect		-4.226	-9.657	1.086
-> MBNMA		-3.490	-7.795	0.466
RT_450 vs Placebo_0	**0.543**			
-> direct		-5.668	-14.578	2.928
-> indirect		-2.698	-6.780	1.597
-> MBNMA		-3.231	-7.148	0.428
RT_400 vs Placebo_0	**0.606**			
-> direct		-1.410	-8.761	6.064
-> indirect		-3.949	-8.422	0.525
-> MBNMA		-3.172	-7.029	0.420
RT_300 vs Placebo_0	**0.565**			
-> direct		-5.094	-12.647	2.950
-> indirect		-2.487	-6.546	1.534
-> MBNMA		-3.006	-6.657	0.391
MBE_550 vs Placebo_0	**0.186**			
-> direct		0.023	-3.942	4.555
-> indirect		-5.133	-9.074	-1.270
-> MBNMA		-2.621	-5.899	0.665
AE-RT_700 vs Placebo_0	**0.264**			

|-> direct		5.022	-2.077	11.863
|-> indirect		-1.624	-6.644	3.558
|-> MBNMA		0.701	-3.679	5.212
AE-RT_550 vs Placebo_0	**0.764**			
|-> direct		-0.200	-7.876	7.922
|-> indirect		1.252	-4.337	6.753
|-> MBNMA		0.681	-3.588	5.019
AE-RT_200 vs Placebo_0	**0.722**			
|-> direct		0.033	-7.478	7.750
|-> indirect		0.768	-3.547	5.018
|-> MBNMA		0.554	-2.961	4.228
AE_1100 vs Placebo_0	**0.285**			
|-> direct		0.881	-16.822	17.721
|-> indirect		-6.191	-9.984	-2.514
|-> MBNMA		-5.981	-9.560	-2.450
AE_750 vs Placebo_0	**0.512**			
|-> direct		-3.275	-10.599	4.184
|-> indirect		-6.560	-10.595	-2.351
|-> MBNMA		-5.804	-9.230	-2.391
AE_600 vs Placebo_0	**0.573**			
|-> direct		-4.457	-13.719	4.900
|-> indirect		-5.809	-9.567	-2.185
|-> MBNMA		-5.692	-8.987	-2.331
AE_550 vs Placebo_0	**0.528**			
|-> direct		-4.677	-14.820	5.113
|-> indirect		-5.745	-9.379	-2.323
|-> MBNMA		-5.634	-8.921	-2.316
AE_450 vs Placebo_0	**0.436**			
|-> direct		-3.583	-16.537	8.778
|-> indirect		-5.541	-8.983	-2.088
|-> MBNMA		-5.511	-8.721	-2.282
AE_350 vs Placebo_0	**0.275**			
|-> direct		-9.273	-15.804	-2.570
|-> indirect		-4.149	-7.578	-1.051
|-> MBNMA		-5.319	-8.447	-2.194

Note: Treatment labels are followed by weekly dose in MET-min/week. AE: Aerobic Exercise; AE-RT:Aerobic and resistance exercise combined training; MBE: Mind-Body Exercise; RT: Resistance Training.

5.3 Dose-response model selection

For model selection, we adopted a quadratic function model. In accordance with the default settings in MBNMAdose, we applied vague (non-informative) prior distributions to minimize bias and allow the data to drive the results, thereby enabling the model to adapt flexibly to the underlying data structure. This approach is designed to capture a specific form of non-linear dose–response relationship and to identify an optimal dose under the assumption of a quadratic association. Below, we present a comparison of parameter estimates across different models (Table S5). In general, lower DIC indicate a better model fit [20]. As the quadratic function model exhibited the lowest DIC value, we selected it for our analysis.

Table S5. Model fit comparison.

Model	DIC	SD	Deviance	Residual deviance	pD
Emax (random treatment effects)	178.6	0.528	140.062	42.700	38.9
Restricted cubic spline (random treatment effects; 3 knots)	182.9	0.567	140.942	43.580	42.2
Log Linear (random treatment effect)	179.8	0.532	140.280	42.918	39.3
Quadratic function (random treatment effects)	178.2	0.528	140.186	42.824	39.6

Note: DIC: deviance information criterion; pD: effective number of parameters; SD: standard deviation.

We also evaluated the fit of the quadratic dose–response model to the data. Figures S4 and S5 present the results of the split network meta-analysis across different weekly exercise doses (MET-min/week) for the overall exercise intervention and individual exercise modalities, with neuropsychiatric symptom scores as the outcome. This analysis aimed to explore the dose–response pattern and determine which functional form best represented the observed data, thereby informing model selection for the subsequent MBNMA. The model that most closely matched the patterns shown in Figures S4 and S5 was selected for the corresponding MBNMA analyses.

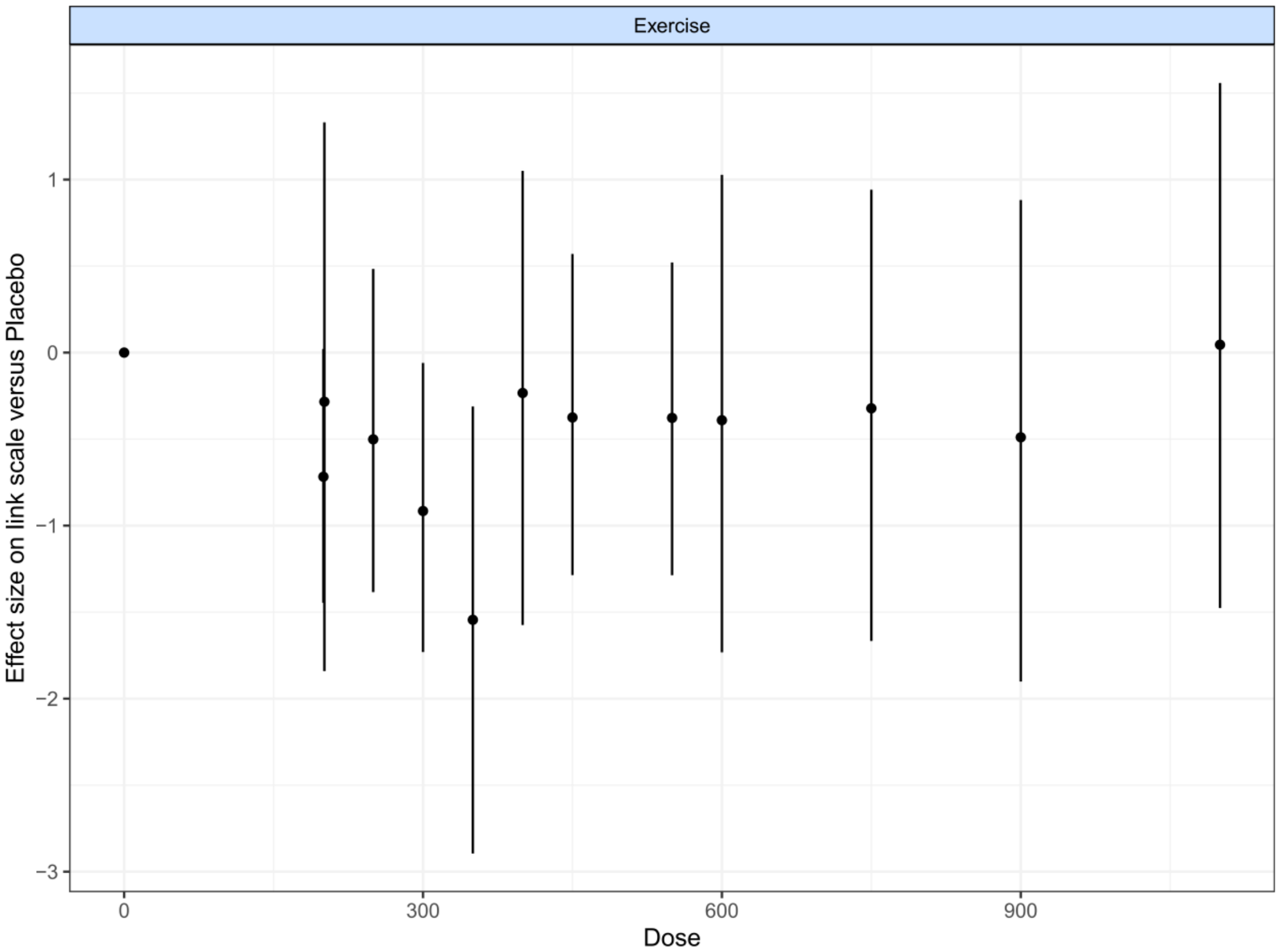

Figure S4. Node-splitting network meta-analysis of exercise interventions for neuropsychiatric symptoms in older adults with cognitive impairment or dementia.

The black dots represent the pooled effect size from the meta-analysis at that dose, and the black vertical lines represent the range of the 95% CrI.

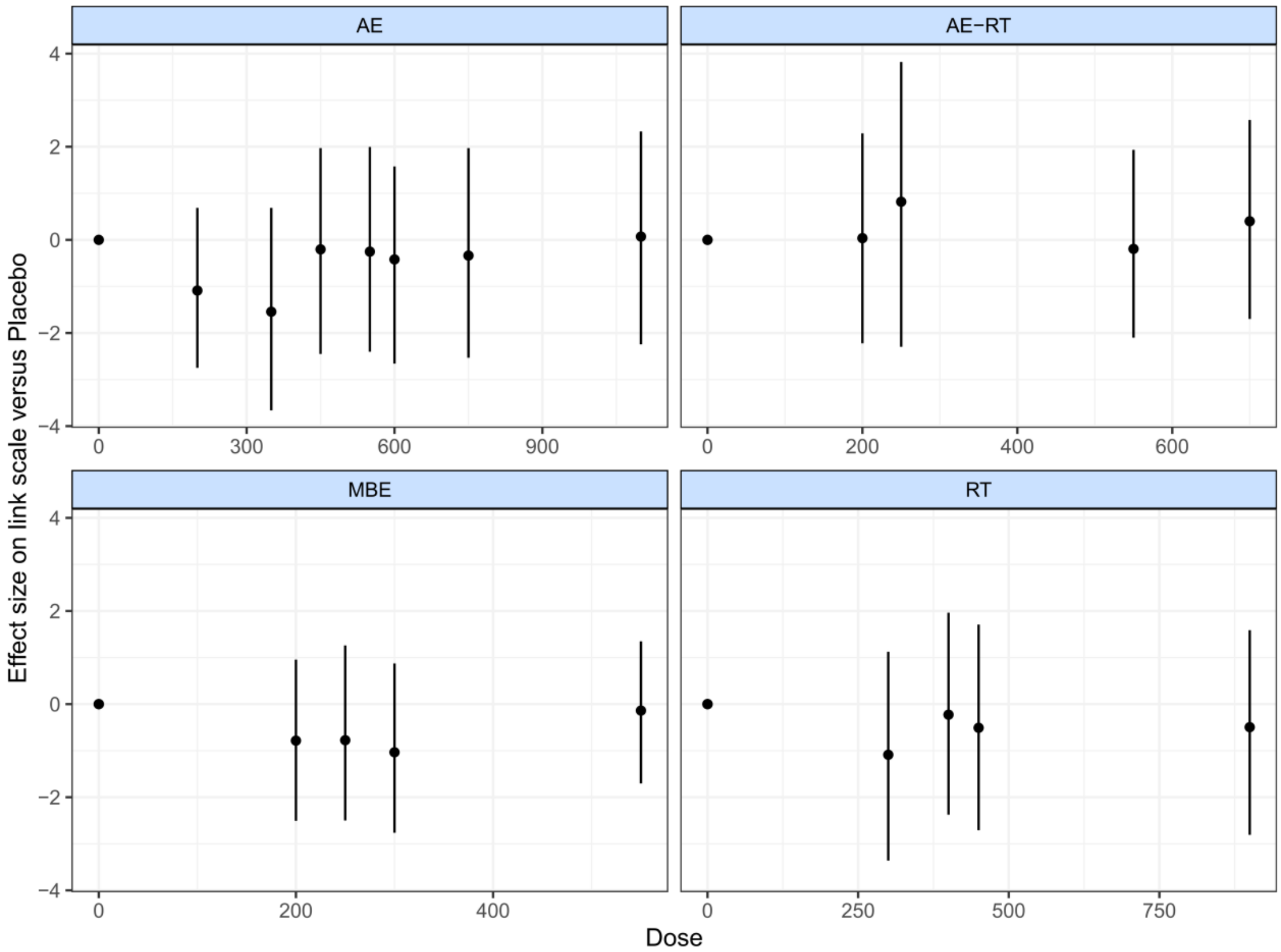

Figure S5. Node-splitting network meta-analysis of different exercise modalities for neuropsychiatric symptoms in older adults with cognitive impairment or dementia.

The black dots represent the pooled effect size from the meta-analysis at that dose, and the black vertical lines represent the range of the 95% CrI. AE: Aerobic Exercise; AE-RT:Aerobic and resistance exercise combined training; MBE: Mind-Body Exercise; RT: Resistance Training.

We observed that the trend of the model curve is generally consistent with the data points from the "Split" network meta-analysis, suggesting a good fit between the model and the data.

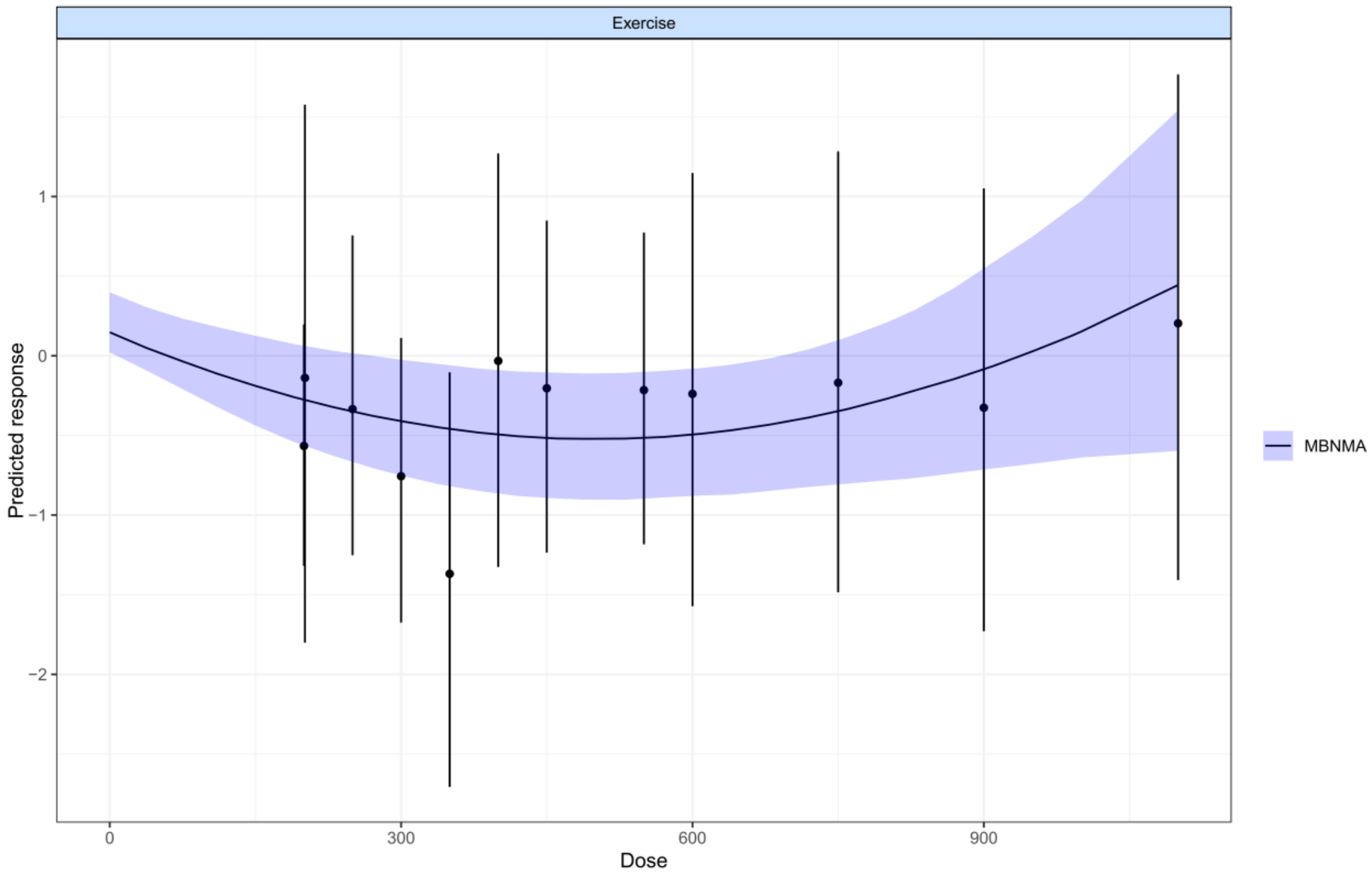

Figure S6. Dose-response relationship between overall exercise dose and neuropsychiatric symptoms in older adults with cognitive impairment or dementia (quadratic function).

The black dots represent the pooled effect size from the meta-analysis at that dose, while the blue region represents the 95% CrI range.

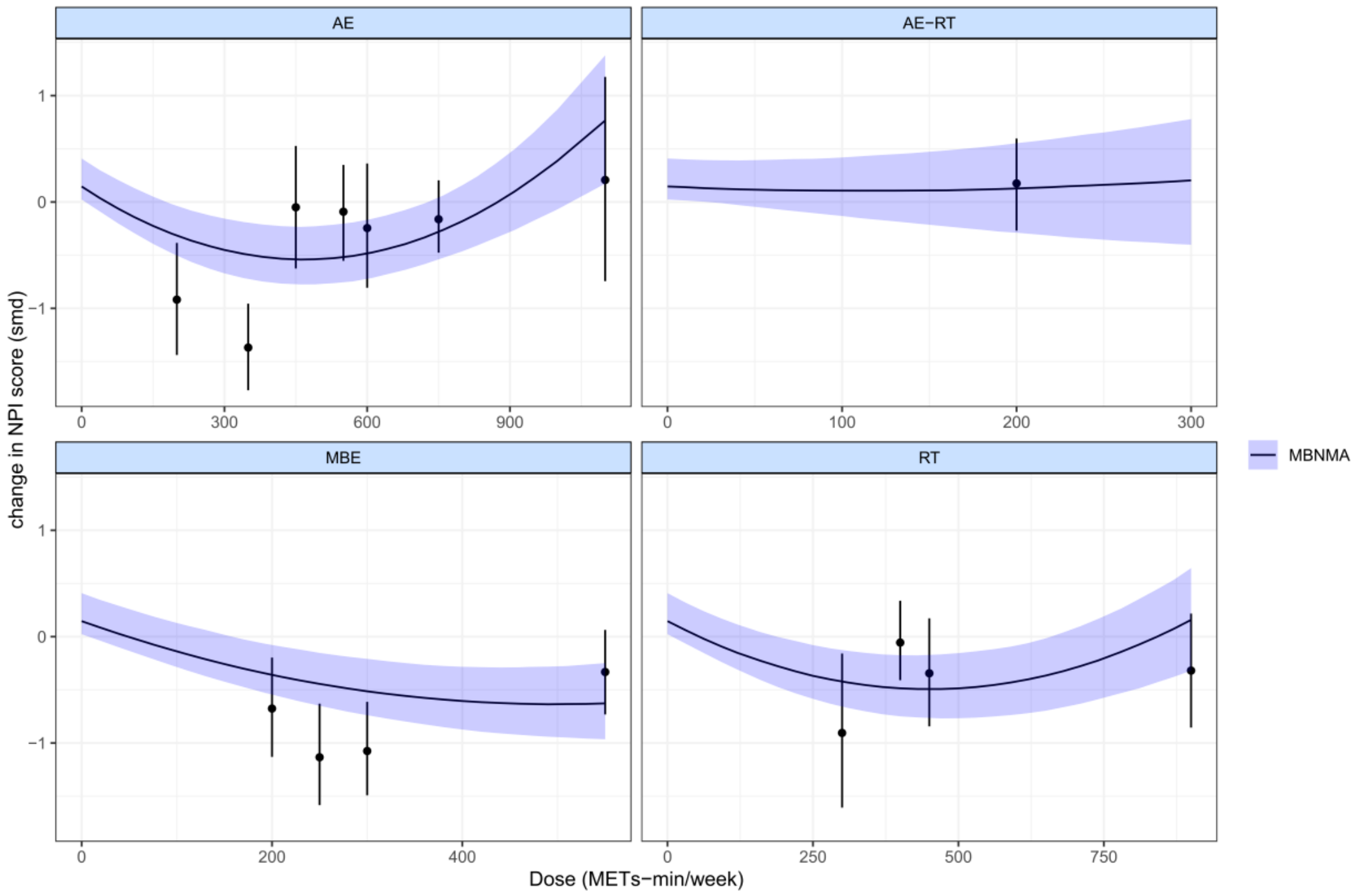

Figure S7. Dose-response relationships between different exercise modalities and neuropsychiatric symptoms in older adults with cognitive impairment or dementia (quadratic function).

The black dots represent the pooled effect size from the meta-analysis at that dose, while the blue region represents the 95% CrI range. AE: Aerobic Exercise; AE-RT:Aerobic and resistance training; MBE: Mind-Body Exercise; RT: Resistance Training.

5.4 Dose-response relationships

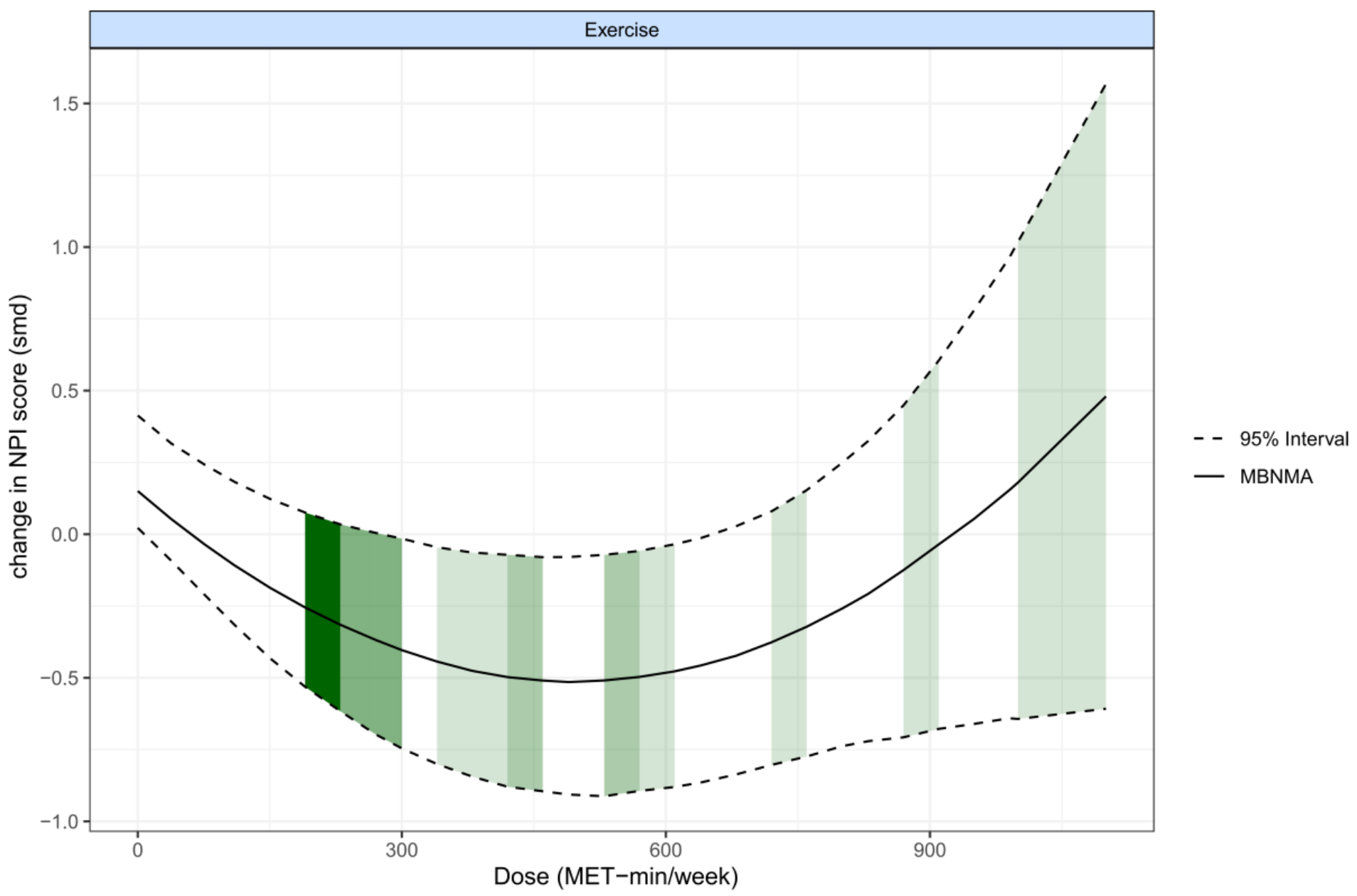

Figure S8. Dose-response relationship between overall exercise dose and neuropsychiatric symptoms in older adults with cognitive impairment or dementia. The green area represents the original study data, with darker shading indicating a greater density of observations.

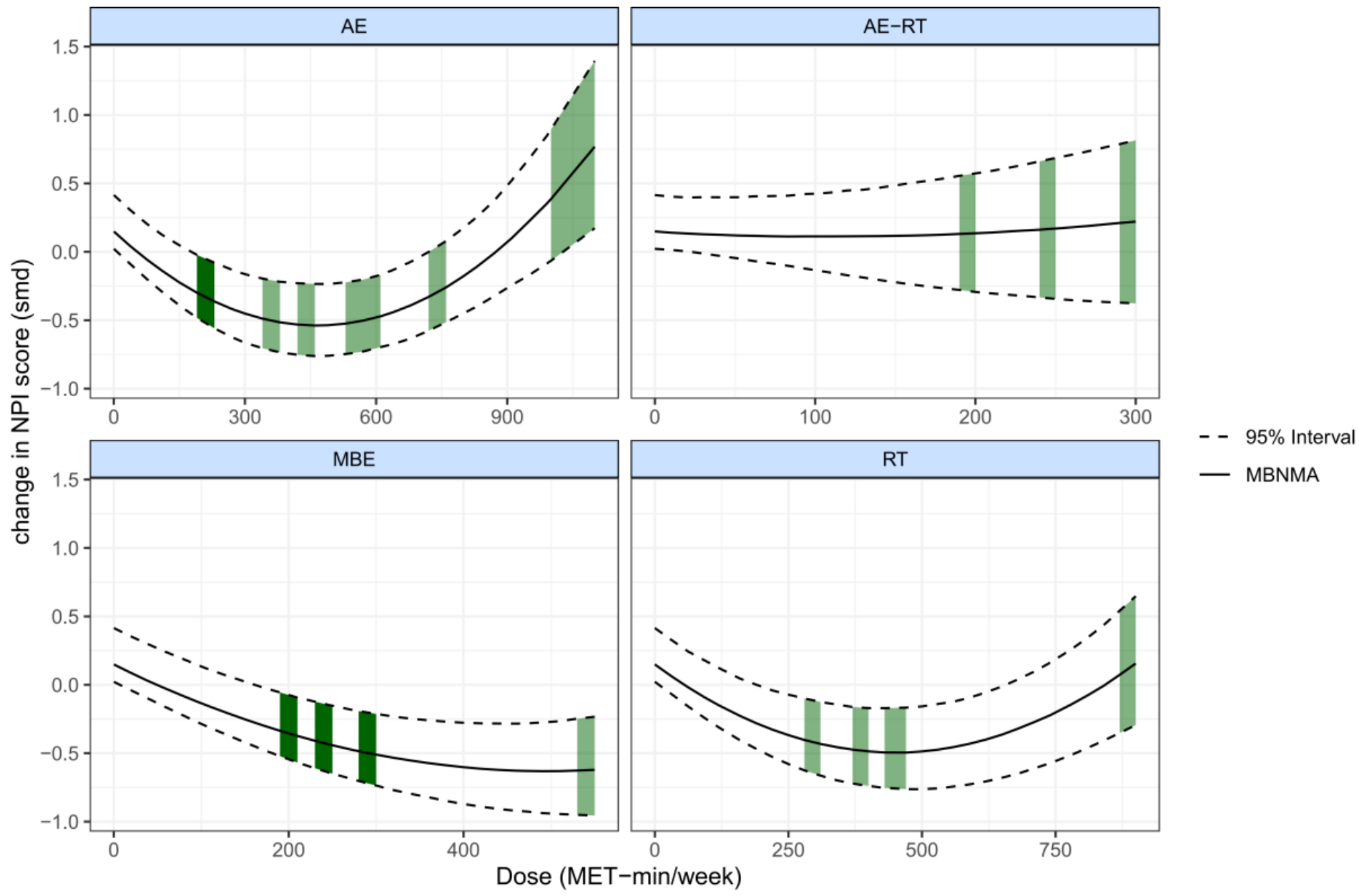

Figure S9. Dose-response relationships between different exercise modalities and neuropsychiatric symptoms in older adults with cognitive impairment or dementia.

(the green area represents the original study dataset; the darker the color, the larger the amount of data)

AE: Aerobic Exercise; AE-RT:Aerobic and Resistance training; MBE: Mind-Body Exercise; RT: Resistance Training.

5.5 Sensitivity analysis

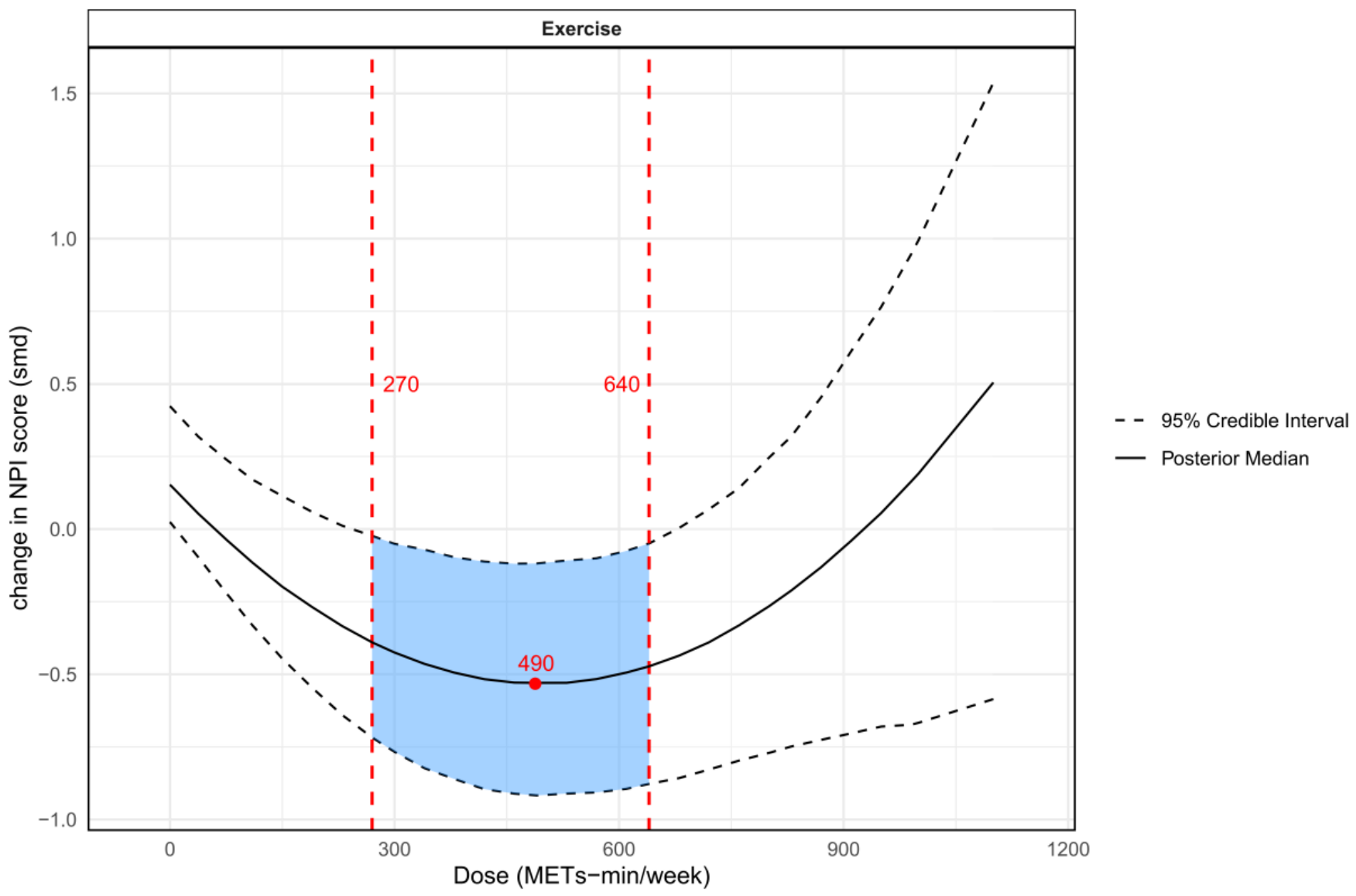

Figure S10. Dose-response relationship between overall exercise dose and neuropsychiatric symptoms in older adults with cognitive impairment or dementia in the sensitivity analysis excluding studies at high risk of bias.

The red dashed vertical lines indicate the model-identified effective dose range from 270 to 640 MET-min/week. The red dot marks the optimal dose at 490 MET-min/week.

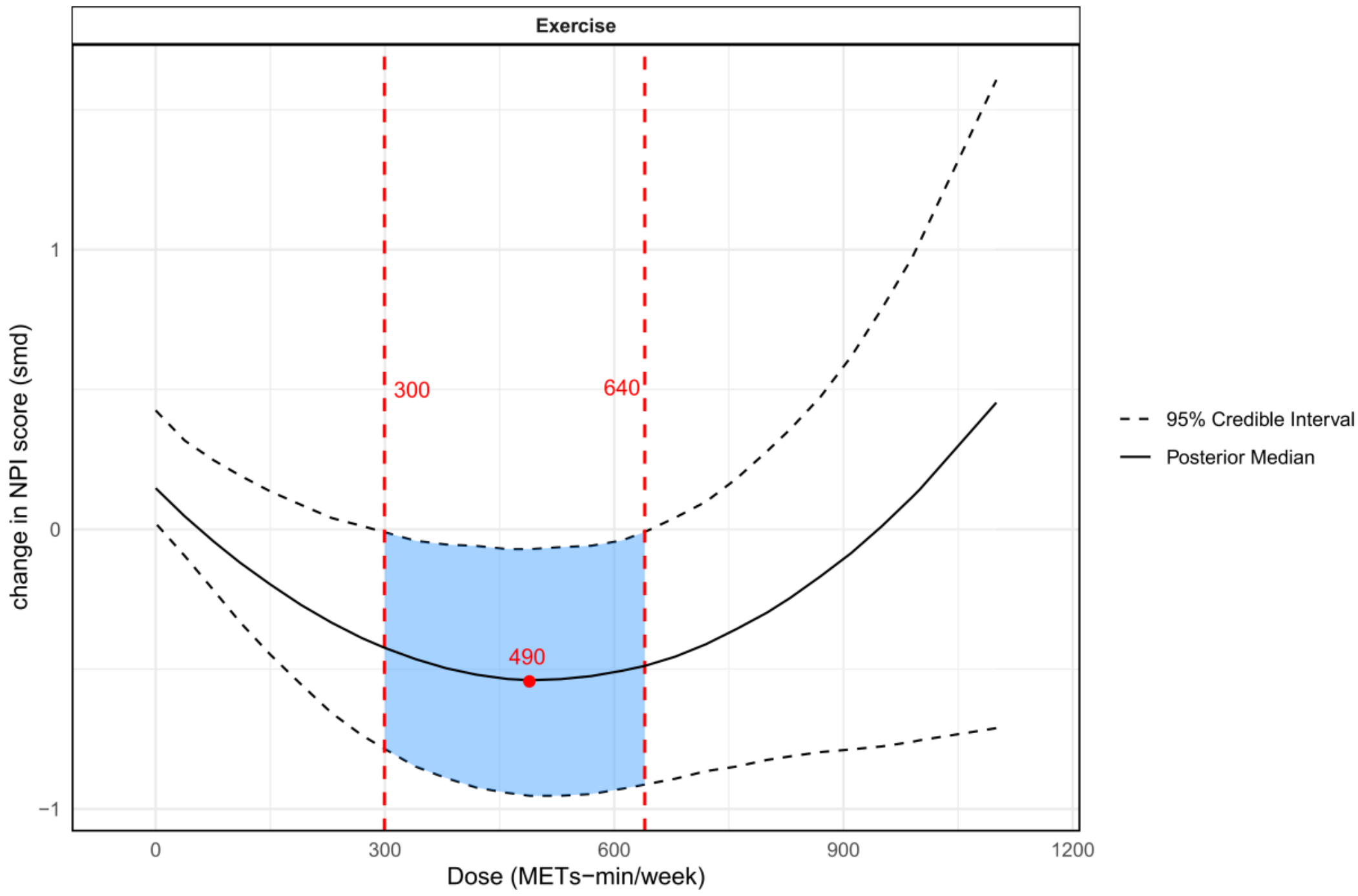

Figure S11. Dose-response relationship between overall exercise dose and neuropsychiatric symptoms in older adults with dementia in a sensitivity analysis excluding studies enrolling participants with mild cognitive impairment.

The red dashed vertical lines indicate the model-identified effective dose range from 300 to 640 MET-min/week. The red dot marks the optimal dose at 490 MET-min/week.

5.6 Relative effectiveness ranking

Table S6. Relative effectiveness rankings across intervention modes and doses.

Rank	Treatment	Mean	Median	2.50%	97.50%
1	MBE_490	9.474333333	3	1	40
2	MBE_510	8.758	4	1	37
3	MBE_470	8.626333333	5	1	34
4	MBE_530	10.73366667	5	1	43
5	MBE_460	9.021	6	1	32
6	MBE_440	9.823333333	7	2	30
7	MBE_550	12.49266667	7	1	46.025
8	MBE_420	11.03633333	8	4	30
9	MBE_400	12.568	9	6	30
10	MBE_380	14.34266667	10	9	32
11	MBE_360	16.35166667	11	11	35
12	AE_460	15.42133333	15	1	35
13	AE_490	16.07433333	16	1	37
14	MBE_340	18.53466667	16	12	38
15	AE_420	17.13933333	17	2	36
16	AE_530	18.75666667	18	2	41
17	MBE_320	21.05866667	19	13	40
18	AE_380	20.591	20	4	39
19	RT_430	20.835	21	2	39
20	RT_470	20.801	21	1	42
21	AE_570	22.74266667	22	4	45
22	MBE_300	23.923	22	14	43
23	RT_500	22.50366667	23	1	45
24	RT_400	22.67633333	24	4	39
25	AE_340	25.19033333	25	6	43
26	MBE_280	27.242	26	15	45
27	RT_530	25.24233333	26	2	48
28	AE_610	27.907	27	6	49
29	RT_370	25.63566667	27	7	41
30	MBE_270	29.59533333	29	16	48
31	RT_560	28.63833333	30	4	52
32	AE_300	30.99533333	31	10	47
33	RT_340	29.36733333	31	9	44
34	AE_640	32.62366667	34	8	53
35	MBE_250	33.57866667	34	17	51
36	RT_310	33.69033333	35	13	47
37	RT_590	32.52433333	35	5	55
38	AE_270	36.39266667	37	16	52
39	MBE_230	37.99266667	39	18	54
40	RT_620	36.882	39	8	60
41	RT_280	38.435	40	18.975	51

42	AE_680	39.174	41	10	59
43	MBE_210	42.64133333	44	25	57
44	RT_250	43.55766667	44	26	55.025
45	RT_650	41.574	44	10	66
46	AE_230	43.94133333	45	27	57
47	MBE_190	47.27666667	48	30	60
48	RT_220	48.64233333	48	36	60
49	RT_680	46.578	48	13	71.025
50	AE_720	46.29266667	49	18	66
51	AE_190	51.55766667	52	38	63
52	MBE_170	51.75133333	52	36	64
53	RT_190	53.70233333	53	46	65
54	RT_710	51.788	53	19	79
55	AE_760	53.66433333	55	24	76
56	MBE_150	56.06366667	56	44	68
57	RT_160	58.72633333	58	52	72
58	RT_740	57.25166667	58	23	91.025
59	AE_150	58.95466667	59	50	72
60	MBE_130	60.24733333	60	50	75
61	AE_800	61.48366667	62	31	92
62	MBE_110	64.39733333	63	55	81
63	RT_120	65.47766667	64	59	82
64	RT_780	65.12933333	64	30.975	105.025
65	AE_110	66.45	65	58	83.025
66	MBE_95	67.983	67	59	86
67	AE_830	68.36666667	67.5	36	104
68	RT_93	70.914	69	63	89
69	RT_810	72.05966667	69	39	109
70	MBE_76	72.47933333	71	63	92
71	AE_76	73.89933333	72	65	94
72	AE_870	78.41633333	75	47	111
73	MBE_57	77.434	75	67.975	98
74	RT_62	77.93933333	75	68	98
75	RT_840	79.35366667	75	46.975	112
76	MBE_38	82.95066667	78	71	104
77	AE_38	84.004	79	72	105
78	RT_31	87.089	81	74	107
79	RT_870	86.88933333	82	53	114
80	MBE_19	90.59433333	83	75	110
81	AE_910	89.59866667	90	56	112
82	AE-RT_120	90.38666667	91	79	98
83	AE-RT_130	90.21466667	91	78	99
84	AE-RT_140	90.108	91	76	100
85	AE-RT_160	89.984	91	73	101

86	AE-RT_170	90.142	91	71.975	102
87	AE-RT_62	93.63733333	92	81	106
88	AE-RT_72	92.83433333	92	81	105
89	AE-RT_83	92.091	92	81	103
90	AE-RT_93	91.49733333	92	80.975	101
91	AE-RT_100	91.091	92	80	100
92	AE-RT_110	90.67333333	92	79	98
93	AE-RT_180	90.377	92.5	69	104
94	AE-RT_52	94.55966667	93	81	108
95	AE-RT_190	90.73433333	94	67	105
96	AE-RT_41	95.608	96	81	110
97	AE-RT_200	91.15133333	96	65	106
98	AE-RT_31	96.61533333	98	81	112
99	AE-RT_210	91.65366667	98	63	107
100	RT_900	94.32133333	99	59	115
101	AE-RT_21	97.633	100	80	113
102	AE-RT_220	92.22833333	100	61	108
103	AE-RT_230	92.90866667	101	59	109
104	AE-RT_10	98.67166667	102	80	114
105	AE-RT_240	93.67533333	103	56	110
106	Placebo_0	99.70433333	104	79	115
107	AE-RT_250	94.522	104	55	111
108	AE-RT_260	95.41733333	106	53	112
109	AE_950	99.38633333	107	64	113
110	AE-RT_270	96.36933333	107	50	113
111	AE-RT_280	97.37466667	109	48	114
112	AE-RT_290	98.44433333	110	46	115
113	AE-RT_300	99.56066667	111	44	116
114	AE_990	106.5046667	114	71	114
115	AE_1000	108.5826667	115	74	115
116	AE_1100	114.823	116	100	116

Note: The number that follows the exercise intervention indicates the weekly exercise dose (MET-min/week). AE: Aerobic Exercise; AE-RT:Aerobic and resistance training; MBE: Mind-Body Exercise; RT: Resistance Training.

6. Meta-regression analysis

Table S7. Dose-response network meta-regression.

Covariate	DIC	Residual Deviance	Shared beta (median and 95% CrI)	SD (median and 95% CrI)
Age (years)	163.4	39.0	-0.074 (-0.555, 0.321)	3.21 (1.86, 5.77)
Female percent (%)	170.1	40.9	-0.045 (-0.189, 0.093)	3.18 (1.93, 5.63)
Intervention length (weeks)	184.5	43.4	-0.043 (-0.152, 0.058)	2.92 (1.72, 4.96)

Note: DIC: deviance information criterion; SD: standard deviation

7. Reporting bias and small-study effects

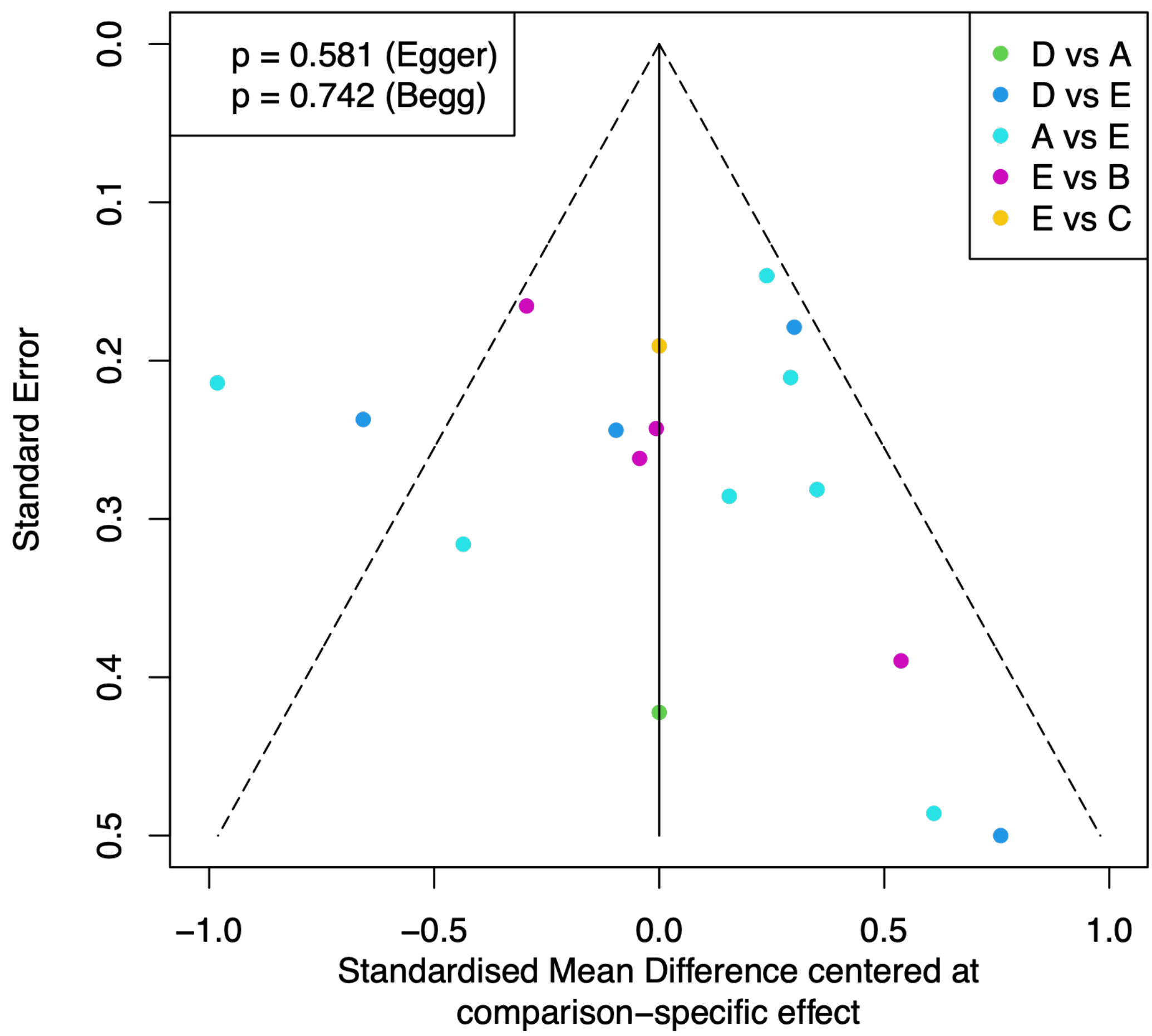

Figure S12. Comparison-adjusted funnel plot.

A: aerobic exercise; B: resistance training; C: aerobic and resistance training; D: mind-body exercise; E: control group

8. Confidence in the evidence using CINeMA

Following the CINeMA guidance (https://cinema.ispm.unibe.ch/), we assessed confidence in the evidence for the effects of different exercise modalities and weekly exercise doses on neuropsychiatric symptoms in older adults with cognitive impairment or dementia. Neuropsychiatric symptoms were assessed using the Neuropsychiatric Inventory (NPI), NPI-Q, NPI-C, or NPI-NH. Higher scores indicate more severe neuropsychiatric symptoms. Each network estimate was evaluated across six CINeMA domains.

8.1 Within-study bias

The risk of bias of each included randomized controlled trial was assessed using the Cochrane Risk of Bias 2 (RoB 2) tool and classified as low risk, some concerns, or high risk. CINeMA contribution matrices were used to determine the contribution of each study to each network estimate.

Exercise interventions cannot generally be double-blinded. Therefore, the absence of participant or therapist blinding was not considered sufficient to classify a study as having high risk of bias. Particular attention was paid to randomization, deviations from intended interventions, missing NPI data, blinding of NPI assessors or proxy reporters, and selective reporting of NPI versions or assessment time points.

Downgrading was based on the risk of bias of the studies contributing most substantially to each network estimate. No concerns were assigned when the evidence was predominantly contributed by studies at low risk of bias, some concerns were assigned when important contributions came from studies with some concerns, and major concerns were assigned when the estimate was substantially influenced by studies at high risk of bias.

8.2 Reporting bias

To minimize the risk of publication and selective reporting bias, we searched the reference lists of relevant systematic reviews and included studies. Reporting bias was assessed using comparison-adjusted funnel plots and Begg’s or Egger’s tests when a sufficient number of studies were available.

Statistical test results were interpreted together with funnel-plot asymmetry, study size, the distribution of exercise modalities and doses, and evidence of selective reporting of NPI outcomes, NPI versions, or follow-up time points. The domain was not downgraded when no meaningful evidence of publication bias or selective outcome reporting was identified.

8.3 Indirectness

Indirectness was assessed according to the clinical and methodological relevance of the included studies to the review question.

Eligible studies included older adults with mild cognitive impairment, Alzheimer’s disease, vascular dementia, mixed dementia, or other major neurocognitive disorders diagnosed according to recognized diagnostic criteria or by qualified clinicians.

The interventions included aerobic exercise, walking, cycling, resistance training, Tai Chi, dance, multicomponent exercise, functional training, exergaming, and other structured exercise modalities. Comparators included no-exercise controls, usual care, health education, waiting-list controls, social activities, cognitive training, stretching or toning exercise, and other active exercise interventions.

Neuropsychiatric symptoms were assessed using NPI-family instruments. Differences in NPI version, reporter or assessor, scoring method, baseline symptom severity, dementia diagnosis and severity, care setting, exercise modality, weekly exercise dose, intervention duration, frequency, session duration, intensity, supervision, and adherence were considered when judging indirectness and the plausibility of the transitivity assumption.

Weekly exercise dose was expressed as MET-min/week. When the original study did not provide a directly quantifiable dose, the dose was estimated from the reported exercise frequency, session duration, and intensity. The uncertainty associated with estimated dose values was considered when interpreting indirectness.

8.4 Imprecision

Neuropsychiatric symptoms were treated as a continuous outcome. The 95% credible intervals were examined to determine whether they crossed the null effect threshold of 0 and whether they included values compatible with both clinically important improvement and little or no improvement.

Because no single minimal clinically important difference has been established across all NPI versions, dementia populations, exercise modalities, and comparisons, the null threshold of 0 was used as the primary statistical decision threshold. The clinical importance of imprecise estimates was interpreted cautiously, considering the NPI score range and the direction of change, in which lower scores indicate improvement.

8.5 Heterogeneity

Heterogeneity was assessed by considering the between-study variance and the extent to which credible and prediction intervals could lead to different clinical conclusions.

Potential sources of heterogeneity included differences in dementia diagnosis and severity, baseline NPI scores, care setting, age, concomitant psychotropic medication, exercise modality, weekly exercise dose, intervention duration, frequency, session duration, intensity, supervision, adherence, and NPI reporter or assessment procedure.

Comparisons or dose-specific estimates were downgraded when heterogeneity was sufficiently large to reduce confidence in the estimated effect or to produce materially different conclusions across studies.

8.6 Incoherence

Incoherence between direct and indirect evidence was assessed using appropriate local and global methods. Statistical significance was considered supporting evidence rather than the sole basis for judgment.

The magnitude and uncertainty of disagreement between direct and indirect estimates were also examined, together with differences in participant characteristics, dementia severity, exercise modality, comparator type, dose category, NPI version, and outcome assessment procedure.

For dose-response estimates, the consistency between the model-based dose-response relationship and the observed data across exercise doses and modalities was also considered. Each network estimate was classified as having no concerns, some concerns, or major concerns for incoherence according to the extent to which disagreement between direct and indirect evidence could alter the interpretation of the estimated effect.

8.7 Overall CINeMA judgment

CINeMA evaluates confidence in each network estimate across six domains: within-study bias, reporting bias, indirectness, imprecision, heterogeneity, and incoherence.

These domain-level judgments were integrated to classify the overall confidence in each comparison or dose-response estimate as high, moderate, low, or very low. Domains were considered jointly to avoid duplicate downgrading when the same underlying limitation affected more than one domain. Overall confidence was downgraded only when the relevant concern was likely to materially influence the interpretation of the network estimate. A lack of statistically significant evidence alone was not considered sufficient to classify the evidence as low or very low confidence.

Table S8. Confidence in the evidence using CINeMA for neuropsychiatric symptom outcomes in older adults with cognitive impairment or dementia.

Comparison	Within-study bias	Reporting bias	Indirectness	Imprecision	Heterogeneity	Incoherence	Confidence rating	Reason(s) for downgrading
**AE:CG**	Some concerns	Low risk	No concerns	No concerns	Major concerns	No concerns	**Very low**	["Within-study bias","Heterogeneity"]
**AE-RT:CG**	Some concerns	Low risk	No concerns	Major concerns	No concerns	Major concerns	**Very low**	["Within-study bias","Imprecision","Incoherence"]
**MBE:CG**	Some concerns	Low risk	No concerns	No concerns	Some concerns	No concerns	**Low**	["Within-study bias","Heterogeneity"]
**RT:CG**	Some concerns	Low risk	No concerns	No concerns	Major concerns	Major concerns	**Very low**	["Within-study bias","Heterogeneity","Incoherence"]

Note: AE: Aerobic Exercise; AE-RT: Aerobic and resistance training; MBE: Mind-Body Exercise; RT: Resistance Training; CG: Control Group

References

1. Ayari S, Abellard A, Sakrani S, et al. Comparison of dance and aerobic exercise on cognition and neuropsychiatric symptoms in sedentary older adults with cognitive impairment. *Eur Geriatr Med*. 2023;14(6):1289-1299. doi:10.1007/s41999-023-00849-z
2. Bennett CG, Guttmann RP, Hackney ME, Amin R, Weaver S. Impacts of adapted dance on mood and physical function among persons living with Alzheimer's disease. *Journal of Alzheimer's Disease*. 2025;105(4):1148-1159. doi:10.1177/13872877241298529
3. Budak M, Bayraktaroglu Z, Hanoglu L. The effects of repetitive transcranial magnetic stimulation and aerobic exercise on cognition, balance and functional brain networks in patients with Alzheimer's disease. *Cogn Neurodyn*. 2023;17(1):39-61. doi:10.1007/s11571-022-09818-x
4. Cámara-Calmaestra R, Martínez-Amat A, Aibar-Almazán A, et al. Resistance exercise to reduce risk of falls in people with Alzheimer's disease: a randomised clinical trial. *Physiotherapy*. 2025;126:101440. doi:10.1016/j.physio.2024.101440
5. Cancela JM, Ayán C, Varela S, Seijo M. Effects of a long-term aerobic exercise intervention on institutionalized patients with dementia. *Journal of Science and Medicine in Sport*. 2016;19(4):293-298. doi:10.1016/j.jsams.2015.05.007
6. D'Amico F, Rehill A, Knapp M, et al. Cost-effectiveness of exercise as a therapy for behavioural and psychological symptoms of dementia within the EVIDEM-E randomised controlled trial. *Int J Geriat Psychiatry*. 2016;31(6):656-665. doi:10.1002/gps.4376
7. Fleiner T, Dauth H, Gersie M, Zijlstra W, Haussermann P. Structured physical exercise improves neuropsychiatric symptoms in acute dementia care: a hospital-based RCT. *Alz Res Therapy*. 2017;9(1):68. doi:10.1186/s13195-017-0289-z
8. Hoffmann K, Sobol NA, Frederiksen KS, et al. Moderate-to-High Intensity Physical Exercise in Patients with Alzheimer's Disease: A Randomized Controlled Trial. *JAD*. 2016;50(2):443-453. doi:10.3233/JAD-150817
9. Holthoff VA, Marschner K, Scharf M, et al. Effects of Physical Activity Training in Patients with Alzheimer's Dementia: Results of a Pilot RCT Study. Quinn TJ, ed. *PLoS ONE*. 2015;10(4):e0121478. doi:10.1371/journal.pone.0121478
10. Huang N, Li W, Rong X, et al. Effects of a Modified Tai Chi Program on Older People with Mild Dementia: A Randomized Controlled Trial. Khalsa DS, ed. *Journal of Alzheimer's Disease*. 2019;72(3):947-956. doi:10.3233/JAD-190487
11. Lam LCW, Chau RCM, Wong BML, et al. A 1-Year Randomized Controlled Trial Comparing Mind Body Exercise (Tai Chi) With Stretching and Toning Exercise on Cognitive Function in Older Chinese Adults at Risk of Cognitive Decline. *Journal of the American Medical Directors Association*. 2012;13(6):568.e15-568.e20. doi:10.1016/j.jamda.2012.03.008
12. Lazarou I, Parastatidis T, Tsolaki A, et al. International Ballroom Dancing Against Neurodegeneration: A Randomized Controlled Trial in Greek

Community-Dwelling Elders With Mild Cognitive impairment. *Am J Alzheimers Dis Other Demen*. 2017;32(8):489-499. doi:10.1177/1533317517725813
13. Maltais M, Rolland Y, Vellas B, et al. Effect of Exercise on Behavioral Symptoms and Pain in Patients With Dementia Living in Nursing Homes. *Am J Alzheimers Dis Other Demen*. 2019;34(2):89-94. doi:10.1177/1533317518803773
14. Rolland Y, Pillard F, Klapouszczak A, et al. Exercise Program for Nursing Home Residents with Alzheimer's Disease: A 1-Year Randomized, Controlled Trial. *J American Geriatrics Society*. 2007;55(2):158-165. doi:10.1111/j.1532-5415.2007.01035.x
15. Swinnen N, Vandenbulcke M, De Bruin ED, et al. The efficacy of exergaming in people with major neurocognitive disorder residing in long-term care facilities: a pilot randomized controlled trial. *Alz Res Therapy*. 2021;13(1):70. doi:10.1186/s13195-021-00806-7
16. Swinnen N, De Bruin ED, Guimarães V, et al. The feasibility of a stepping exergame prototype for older adults with major neurocognitive disorder residing in a long-term care facility: a mixed methods pilot study. *Disability and Rehabilitation*. 2024;46(5):896-910. doi:10.1080/09638288.2023.2182916
17. Telenius EW, Engedal K, Bergland A. Effect of a High-Intensity Exercise Program on Physical Function and Mental Health in Nursing Home Residents with Dementia: An Assessor Blinded Randomized Controlled Trial. Cameron DW, ed. *PLoS ONE*. 2015;10(5):e0126102. doi:10.1371/journal.pone.0126102
18. Todri J, Todri A, Lena O. Why Not a Global Postural Reeducation as an Alternative Therapy Applied to Alzheimer's Patients in Nursing Homes? A Pioneer Randomized Controlled Trial. *Dement Geriatr Cogn Disord*. 2019;48(3-4):172-179. doi:10.1159/000505397
19. Yang SY, Shan CL, Qing H, et al. The Effects of Aerobic Exercise on Cognitive Function of Alzheimer's Disease Patients. *CNSNDDT*. 2015;14(10):1292-1297. doi:10.2174/1871527315666151111123319
20. MBNMAdose: Perform a Model-Based Network Meta-Analysis (MBNMA). Accessed August 8, 2026. https://hugaped.github.io/MBNMAdose/articles/runmbnmadose-2.html#model-selection
